# Isolation and Characterization of *Bifidobacterium longum* A1, an Isolate from Faeces of an Exclusively Breast-fed Infant with Probiotic Potential

**DOI:** 10.1007/s12602-026-11017-7

**Published:** 2026-04-14

**Authors:** Lise Sanchez, Marcus Vinicius Canario Viana, Veronique Robert, Florian Chain, Sead Chadi, Vasco Azevedo, Alexis Mosca, Philippe Langella, Sylvie Binda, Rebeca Martín

**Affiliations:** 1 Micalis Institute, AgroParisTech, INRAE, Université Paris-Saclay, Jouy-en-Josas, France; 2 Lallemand Health Solutions, Montreal, Canada; 3 Université Paris Diderot, INSERM, Robert Debré Hospital, APHP, Paris, France; 4 Department of Genetics, Ecology and Evolution, Institute of Biological Sciences, Federal University of Minas Gerais, Belo Horizonte, Minas Gerais Brazil

**Keywords:** Breast-fed infant, Primocolonization, Bacteria isolation, *Bifidobacterium*, Probiotics, Safety assessment, Genome sequencing, Probiotics

## Abstract

Microbial colonization of the newborn (primocolonization) plays a crucial role in the development of digestive, metabolic, and immune systems. Disruptions may impair intestinal barrier function and raise the risk of chronic diseases like inflammatory bowel disease, allergies, and obesity. Recent evidence suggests probiotic supplementation in infants may help counter or prevent these effects. This study isolates and characterizes potential probiotic candidates from the gut microbiota of exclusively breastfed infants. *Bifidobacterium* and *Lacticaseibacillus* strains were selectively isolated and initially characterized in vitro. To evaluate their ability to reach the colon intact, we assessed their growth and bile tolerance. Strains safety was evaluated in vitro by determining antibiotic resistance profiles, DNase, and β-hemolytic activities. Additionally, their potential effects on the intestinal barrier and their ability to modulate the immune response were assessed in order to highlight potential probiotic functionality. The most promising strains underwent further characterizations. We performed whole genome sequencing (WGS) analysis to examine transferable antibiotic resistance, virulence factors, toxicity, and biogenic amine formation, thereby confirming the safety of the strains. Bile salt hydrolase (BSH) and glycoside hydrolase (GH) genes were also investigated. Furthermore, we explored the ability of certain strains to ferment human milk oligosaccharides (HMOs) and engage in cross-feeding interactions. The study showed that isolated strains exhibit probiotic traits such as acid and bile resistance, immunomodulatory potential, and strong safety with no detected virulence or resistance genes. *Bifidobacterium longum* A1 stood out for its metabolic strength, safety, ability to degrade human milk oligosaccharides, and support beneficial cross-feeding.

**Supplementary Information:**

The online version contains supplementary material available at 10.1007/s12602-026-11017-7.

## Introduction

Gut colonization of the new-born (primocolonization) is a dynamic and complex process mainly driven by mother-to-infant vertical transmission [1–3]. It is generally admitted that this process begins at birth, when the new-born is massively exposed to mother vaginal and gut microbiota during the passage through the birth canal [4, 5]. After birth, the maternal microbial imprint continues through breastfeeding, which facilitates the transfer of microbes present in breast milk and on the mother skin [6, 7]. Breast milk plays a crucial role in the development of the infant gut microbiota, notably due to the presence of human milk oligosaccharides (HMOs). These indigestible carbohydrates, abundant in breast milk, serve as a key energy source for beneficial gut bacteria, particularly for *Bifidobacterium*, *Lactobacillus*, and *Lacticaseibacillus* species [8, 9]. The introduction of solid food marks the beginning of weaning. This natural and complex process leads to a major shift in the infant microbial communities, which diversify in composition and functionality to better meet the evolving nutritional needs of the developing child [10]. Thus, the World Health Organization (WHO) recommends exclusive breastfeeding for the first six months of life, with continued breastfeeding up to two years of age or beyond.

Establishing a healthy early gut microbiota, enriched in beneficial commensal bacteria such as bifidobacteria and lactobacilli, is crucial for infant development, as it it supports metabolic functions adapted to milk-based nutrition, including the production of key microbial metabolites, and contributes to immune and epithelial maturation [11, 12]. Among its many functions, the early microbiota promotes the maturation of the intestinal mucosa, strengthens the epithelial barrier and reduces intestinal permeability [13, 14]. Beneficial microorganisms also occupy ecological niches, thereby limiting pathogen adhesion and proliferation, contributing to protection against infection and inflammation. In addition, the early intestinal microbiota is essential for digesting complex carbohydrates and producing vital metabolites, such as vitamins and short-chain fatty acids (SCFAs) [15–18]. Beyond these direct functions, microbial communities engage in cross-feeding interactions, whereby certain bacterial species utilize metabolites produced by others, contributing to the maturation and stability of the neonatal gut ecosystem [19–21]. Furthermore, the early intestinal microbiota closely interacts with the infant developing immune system, influencing its response to external stimuli [14, 22, 23]. It contributes to the maturation and education of the immune system by regulating the balance between inflammatory responses and promoting immune tolerance [13, 24, 25]. The perinatal period, beginning at birth and extending through the first months of life, therefore represents a critical window during which primary gut colonization and early microbiota maturation enables bidirectional communication between the microbiota and the host, promoting the establishment of intestinal and immune homeostasis.

Major factors associated with Western lifestyle practices, such as mode of delivery, antibiotic use, and infant formula feeding, can disrupt gut microbial assembly. These factors have been identified as major determinants of primary gut colonization and early microbiota development during perinatal period, beginning at birth and extending through the first months of life [26]. Disturbances of maternal microbiota imprinting during this critical period can hinder the establishment of communication between the host and the microbiota, which could compromise the infant healthy development [27, 28]. This may increase susceptibility to Non-Communicable Diseases (NCDs) such as Inflammatory Bowel Disease (IBD), allergies and obesity later in life [22, 29–31].

Probiotic supplementation in infants may help prevent and treat certain NCDs. Emerging evidence highlights the efficacy of probiotics in supporting gastrointestinal health, reducing the risk of allergies, asthma, and antibiotic-associated diarrhoea (AAD), and enhancing immune function in infants [32–35]. The World Health Organization (WHO) and the International Scientific Association for Probiotics and Prebiotics (ISAPP) define probiotics as ‘live microorganisms that, when administered in adequate amount, confer a health benefit on the host [36]. This means that to be considered as probiotics, microorganisms must be safe and have scientifically proven health benefits on the host. In the United States, species or groups of microorganisms considered safe for human and animal consumption are granted “Generally Recognized As Safe” (GRAS) status. Similarly, in Europe, the European Food Safety Authority (EFSA) grants (Qualified Presumption of Safety (QPS) status to such microorganisms. However, these statuses do not guarantee the safety of all strains within these species. For that reason, authorities recommend assessment of each strain individually to ensure it is exempt from harmful characteristics, such as toxin production, antibiotic resistance, or DNase activity.

Among probiotic bacterial strains, species from the genera *Lacticaseibacillus*, *Lactiplantibacillus*, *Limosilactobacillus*, *Lactobacillus* and *Bifidobacterium* have been extensively studied for their potential beneficial effects in infants. Species belonging to these genera are particularly relevant to the health of infants’ microbiota, as infants whose initial colonisation is impaired have lower overall levels of these species in their microbiota [28]. Moreover, species form these genera have been included in EFSA’s QPS list. Certain strains of *Lacticaseibacillus casei* and *Lacticaseibacillus rhamnosus* have been shown to be effective in managing acute gastroenteritis and preventing AAD in children [32]. Additionally, a combination of these two strains offers benefits for children with atopic dermatitis and cow’s milk protein allergy [37]. *Bifidobacterium animalis* subsp. *lactis* BB-12^®^ (BB12) and *Limosilactobacillus reuteri* DSM 17938 also appear to have an effect in managing the symptoms of childhood colitis [38, 39]. Regarding the *Bifidobacterium* genus, clinical trials demonstrated that oral administration of a combination of *Bifidobacterium longum* CECT7894 and *Pediococcus pentosaceus* CECT8330 tended to alleviate symptoms of infantile colic [40]. Also, incorporating BB12 into infant formula mitigated immune-related effects associated with non-breastfeeding and caesarean delivery, and reduced the risk of respiratory tract infections in early childhood [41, 42].

In this project we aimed to isolate *Bifidobacterium*, *Lacticaseibacillus*, *Lactiplantibacillus*, *Lactobacillus*, and *Limosilactobacillus* strains from the stool of healthy, vaginally delivered, exclusively breastfed newborns. The goal was to select potential probiotic candidates that could support newborn development and the optimal intestinal colonization in infants. To assess these potential properties, we performed several tests on these strains to characterize them.

## Materials and Methods

This study was conducted in accordance with the ethical standards outlined in the Declaration of Helsinki. Ethical approval was granted by the *Comité de Protection des personnes* (CPP) Ile de France IV (Hôpital Saint-Louis, IRB number 00003835) on January 17th 2022 (ID-RCB 2021-A02888-33). Prior to inclusion, all participating mothers received detailed information regarding the study objectives and procedures and provided written informed consent.

### Samples Collection

Stool samples were collected from 6 babies based on several inclusion criteria (Supplementary data 1). Samples were collected each month from the age of 1 to 6 months. Only the samples from months 1, 3 and 5 were used for the isolation (Supplementary data 2). Mothers were in charge of collecting the uppermost layer of faecal matter in infant’s diapers and placing it into the GutAlive^®^ (MicroViable Therapeutics SL, Gijón, Asturias, Spain) sampling device according to the manufacturer instructions to maintain anaerobic conditions. Samples were treated in the laboratory within 4 days following the collection.

### Bacteria Isolation

Bacteria were isolated in a Coy chamber (Coy Laboratory Products, Grasse Lake, MI, USA) in an anaerobic atmosphere (5% H_2_, 5% CO_2_, 90% N_2_). 10-fold serial dilutions of fecal material were plated onto solid deMan, Rogosa and Sharpe medium, (MRS; BD Difco, Franklin Lakes, NJ, USA) in order to select the targeted groups (bifidobacterial and lactobacilli). Following 48 h of incubation, an average of 10 bacterial isolates per stool samples were inoculated to their corresponding liquid media and reincubated at 37 °C for 24 h. A secondary subculture was performed with 1% (v/v) inoculum under the same growth conditions. After 24 h of incubation, cultures were aliquoted and stored at −80 °C in medium with 20% (v/v) glycerol.

### Identification of the Isolated Strains

Genomic DNA was extracted from each isolate using 200 µL of an overnight culture prepared from cryopreserved stocks. Cultures were pelleted by centrifugation at 2,000 rpm for 5 min and resuspended in 100 µL of sterile Milli-Q water. Bacterial cells were lysed by mechanical disruption with glass beads (0.10–0.25 mm) added to obtain a bead layer of approximately 1 to 2 mm at the bottom of the tube, using a Precellys homogenizer (Bertin Technologies, Montigny-le-Bretonneux, France) at 6,300 rpm for three cycles of 30 s, with a 1-min cooling interval on ice between cycles. The resulting lysate was centrifuged at 2,000 rpm for 5 min, and the supernatant containing genomic DNA was used directly as the template for PCR amplification. The 16S rRNA gene was amplified by using the Dream Taq Polymerase (Thermo Scientific, USA) according to the supplier’s instructions. The amplification targeted the V1 to V9 region of the 16S rDNA gene (1500 bp) using specific primers: (008F) 5’ – GAGTTTGATCCTGGCTCAG – 3’ and (1517R) 5’- ACGGCTACCTTGTTACGGACTT – 3’. The amplified samples were sent to Eurofingenomics (France) for Illumina NovaSeq 6000 sequencing. To identify the obtained sequences, we conducted a search for similarities between the queried sequences (Supplementary Data 3) and those available in the NCBI collection (GenBank, DDBJ, EMBL & PDB) using BLAST (Basic Local Alignment Search Tool; Supplementary Data 4; https://blast.ncbi.nlm.nih.gov/Blast.cgi).

### Bacteria Growth Conditions

To ensure consistency and minimize bias, all bacterial cultures were standardized employing identical preparation methods. From stored cryotubes, strains were cultivated in a Coy anaerobic chamber into MRS medium solidified with 15 g/L of agar (Invitrogen, USA) and supplemented with 0.05% L-cysteine hydrochloride monohydrate (MRSc; Sigma Aldrich, Germany), followed by a 48-hour incubation at 37 °C. Well-isolated colonies were subsequently transferred into MRSc broth medium and incubated anaerobically for 24 h at 37 °C. The cultures were then subcultured (1% v/v) under the same conditions for a further 24 h. The experiments were carried out using bacteria at stationary phase of culture.

*Lacticaseibacillus rhamnosus* GG (LGG) was purchased from the American Type Culture Collection (ATCC 53103) and served as the reference strain for selected experiments. For the co-cultures with eukaryotic cell lines, bacterial cultures were prepared from stationary phase cultures, washed, pelleted, and resuspended in Dulbecco’s Modified PBS (DPBS; Gibco, Thermo Fisher, USA) before use.

### Growth Determinations and Short Chain Fatty Acids (SCFA) Analysis

From a stationary phase preculture, strains were grown at 1% (v/v) inoculum in 50 mL of MRSc and incubated anaerobically (5% H_2_, 5% CO_2_, 90% N_2_) during 24 h at 37 °C. To analyse growth of the strains, Optical Density at a wavelength of 600 nm (OD_600_) was measured every hour during 24 h using a spectrophotometer (Ultrospec 10, Biochrom, Holliston, MA, USA). Supernatant samples were collected once the cultures reached stationary phase and stored at −20 °C for Short Chain Fatty Acids (SCFA) quantification. SCFA content was determined using Gas Chromatography (GC) equipped with Flame Ionization Detection (FID) using the Agilent 7890 GC System (Agilent Technologies, Santa Clara, CA, USA) as previously described [43]. Biological and technical duplicates were used for SCFA quantification.

### *In vitro* Carbohydrates Fermentation

In vitro carbohydrates fermentation was assessed using API 50 CHL System kit (BioMerieux, Marcy-l’Étoile, France) according to the manufacturer’s instructions. From stationary phase cultures, bacterial suspension was prepared in Dulbecco’s Modified PBS (DPBS; Gibco, Thermo Fisher, USA) at 2 McFarland. API galleries were inoculated with 100µL of the bacterial suspension and incubated 48–72 h in anaerobic conditions at 37 °C. A yellow colour change in the inoculated well indicates a decrease in pH, which translates into fermentation of the carbohydrate being tested. The colours ranged from yellow to dark green, reflecting different degrees of carbohydrate fermentation. This assay was performed once.

### Gastro-intestinal Tract Resistance Assessment

Tolerance to bile salts was investigated to simulate the transit of the strains through the gastrointestinal tract. Bacterial stationary-phase cultures were washed, pelleted, and resuspended in PBS before being inoculated at an OD_600_ of 0.1 in 40 mL of fresh MRS medium supplemented with either 0% or 0.3% bovine bile (Oxgall Powder, Sigma Aldrich, France). Resistance to bile salts was analysed by measuring OD_600_ every hour over a period of 12–24 h, as already described. Bacterial stationary phase culture was washed, pelleted and resuspended in 4 ml of PBS at OD_600_ of 1. The 4 ml of bacterial culture were divided in 4 tubes of 1 ml and either PBS or 0.3% bile salt were added, or adjusted at pH3 and pH5. Resistance to bile salt shock was assessed by counting the number of viable cells after 1 h of exposure to either 0% or 0.3% bovine bile, as well as to acidic solutions (pH 3 and pH 6). Numeration was performed by plating 10-fold serial dilutions of the bacterial culture on MRS agar plates, followed by 48 h of incubation at 37 °C. After growth, the number of colony-forming units per millilitre was determined. Biological duplicates were performed for bile salt shock experiment.

### *In vitro* DNase and β-Hemolytic Activity Detection

After culturing the bacteria on solid MRS agar for 48 h at 37 °C, well-isolated colonies were inoculated onto DNase test agar plates supplemented with toluidine blue (Merck Millipore, Germany) and incubated under the same conditions. A clear area around the colonies indicates nuclease activity.

The hemolytic activity of our strain was assessed using blood agar plates (Thermo Fisher, USA), which were streaked with our strain and then incubated at 37 °C for 48 h. Following incubation, the hemolytic activity was evaluated based on the observation of red blood cell lysis in the medium surrounding the colonies.

### Antimicrobial Susceptibility Testing

Phenotypic identification of antimicrobial susceptibility was performed using Minimum Inhibitory Concentration (MIC) determinations, following EFSA guidelines (European Food Safety Authority, 2018). Lactic Acid Bacteria Susceptibility Test Medium (LSM agar), a specialized medium for certain lactic acid bacteria and *Bifidobacterium* species, was prepared with 90% IST (Iso-Sensitest broth, Thermo Scientific, USA), 10% MRS broth (BD Difco, Franklin Lakes, NJ, USA), and 1.5% granulated agar (Selected agar, Invitrogen, USA). A well-isolated colony from MRS plate was suspended in DPBS to a density of 0.5 McFarland and inoculated onto LSM agar using a swab to create a bacterial lawn. Antibiotic strips (BioMerieux, Marcy-l’Étoile, France) were applied, and inhibition zones, along with their corresponding MIC values, were measured and compared to EFSA cut-off values. The following antibiotics were tested for all bacteria: ampicillin, gentamycin, streptomycin, erythromycin, clindamycin, and chloramphenicol. Additionally, vancomycin was tested exclusively for *Bifidobacterium* species, while kanamycin was tested only for *Lacticaseibacillus* species, as recommended by EFSA guidelines. For each species-antibiotic combination, a minimum of two biological replicates were conducted, with two extra replicates specifically included for *L. rhamnosus* strains.

### Eukaryotic Cell Lines Culture Conditions

The HT-29 (ATCC HTB-38) and Caco-2 human (ATCC HTB-37) colon adenocarcinoma cell lines were acquired from the American Type Culture Collection (ATCC, United Kingdom). HT-29 cells were cultured in Dulbecco’s Modified Eagle’s Medium supplemented with GlutaMAX (DMEM; Gibco, Thermo Fisher, USA), 10% (v/v) heat-inactivated foetal bovine serum (FBS; Eurobio, Les Ulis, France) and 0.1% penicillin/streptomycin (Thermo Fisher, USA). The Caco-2 cell line was maintained in the same medium with addition of 1% (v/v) non-essential amino acids. Both cell cultures were incubated at 37 °C with 10% CO_2_, and the medium was renewed every 2 days.

### Anti-inflammatory *in vitro* Assay on HT-29 Cells

HT-29 cells were seeded into 24-well plates at a density of 1 × 10^5 cells per well. The medium was renewed every 2 days with DMEM supplemented with 10% FBS and replaced with a 5% FBS-supplemented DMEM medium on the 6th day. Following a 24-hour incubation period, on day 7, co-incubation with bacterial cells was initiated at a multiplicity of infection (MOI) in DMEM GlutaMAX supplemented with 0.1% penicillin/streptomycin and 5% FBS, with or without the addition of TNF-α at a final concentration of 5 ng/mL (PeproTech, Thermo Fisher, USA). DPBS served as the negative control, while butyrate at 10 mM served as the positive control. After 6 h of co-incubation, supernatants were collected and stored at − 80 °C. Interleukin (IL)−8 concentrations were quantified using the Human IL-8 ELISA MAX Standard Set (BioLegend, San Diego, CA, USA), following the manufacturer’s instructions. Absorbance was measured at 450 nm using an Infinite M200 Pro microplate reader (TECAN, Männedorf, Switzerland). The assays were performed in triplicate with three additional technical triplicates.

### Transepithelial Electrical Resistance Measurements

Caco-2 cells were cultured on Transwell^®^ inserts (Corning™ 3378, France) and maintained at 37 °C with 10% CO₂ until they reached 80% confluence. The culture medium was refreshed every two days until TransEpithelial Electrical Resistance (TEER) values between 2000 and 4000 ohms were achieved. On the day of co-incubation, an initial TEER measurement (T0) was taken before adding bacteria. The test strains or a reference strain, *Lacticaseibacillus rhamnosus* GG [44], were then added to the apical compartment of the insert at a multiplicity of infection (MOI) of 40, while DPBS served as the negative control. After three hours of incubation, 100 ng/mL of TNF-α was introduced into the basal compartment of the Transwell^®^ plates. The plates were then incubated for 24 h before the final TEER measurement (T24) was taken. The results were normalized to basal TEER as follows:

$$\:Ratio=\:\frac{TEER\:treatment\:T24/TEER\:treatment\:T0}{TEER\:negative\:control\:T24/TEER\:\:negative\:control\:T0}$$

The assays were performed in triplicate with three additional technical triplicates.

### Utilization of Human Milk Oligosaccharides (HMO)

The ability to utilize Human Milk Oligosaccharides (HMOs) was assessed by analysing growth and metabolite production of the selected strains in a semi-defined MRS medium [45]. Modified MRS (MRSm) medium was prepared with trypticase peptone (10 g/l), granulated yeast extract (2.5 g/l), tryptone (3 g/l), K_2_HPO_4_ (3 g/l), KH_2_PO_4_ (3 g/l), triammonium citrate (2 g/l), pyruvic acid (0.2 g/l), cysteine HCl (0.3 g/l), Tween-80 (1 ml), MgSO_4_.7H_2_O (0.575 g/l), MnSO_4_.4H_2_O (0.12 g/l), and FeSO_4_.7H_2_O (0.034 g/l). The pH of the medium was adjusted to 6.8 before autoclaving (121 °C for 15 min) and supplemented with 0.5% L-cysteine post-autoclaving.

Strains were inoculated at 1% from an overnight culture into 12-well plates and cultured in mMRS medium supplemented with either no glucose, 0.5% (w/v) glucose (Sigma Aldrich) or 0.5% (w/v) of individual HMOs − 2’-fucosyllactose (2’FL), 3-fucosyllactose (3FL), 3’-sialyllactose (3’SL), 6’-sialyllactose (6’SL) or Lacto-N-tetraose (LNT) - purchased from Elicityl (France). Growth was monitored by recording OD_600_ variations using a Stratus microplate reader (Cerillo, USA). As the instrument reports OD₆₀₀ as a relative optical density normalized to the baseline measurement, the values obtained represent relative OD changes rather than absolute OD₆₀₀ values. SCFA concentrations were quantified in the supernatant of the cultures during the stationary phase using the same method as previously described.

### Genomic Characterization

Genomic DNA was extracted from 15 mL of bacterial culture. To obtained bacterial intra cellular content, bacterial wall was lysed using an enzymatic cocktail consisting of mutanolysin at 233.3 U/mL, lysostaphin at 13.3 U/mL and lysozyme at 100 mg/mL, followed by treatment with RNAse A (Thermo Fisher, USA) at 10 mg/mL and proteinase K (Euromedex, Souffelweyersheim, France) at 50 mg/mL. DNA extraction was carried out on the following supernatant, using Genomic DNA Buffer Set (Qiagen, Hilden, Germany) and the purification by using the Genomic Tips 100/G (Qiagen, Hilden, Germany) following the manufacturer’s instructions. The DNA was resuspended in TE buffer, and its concentration was measured using NanoDrop (NanoDrop 1,000, Thermo Fisher, USA).

The genomes were sequenced using the PacBio VEGA platform (Pacific Biosciences) at the GeT-PlaGe core facility (GenoToul, INRAE, Toulouse, France), using a HiFi SMRTbell library prepared according to the manufacturer instructions. The sequencing reads were trimmed using Fastlplong v.0.2.2 [46] and *de novo* assemblies were performed using Flye v.2.9.5 [47]. The assemblies were evaluated for fragmentation using QUAST v.5.2.0 [48], completeness and contamination using CheckM2 v.1.0.2 [49] and GUNC v.1.0.6 [50], and for taxonomy using GTDB-Tk v.2.4.0 [51] with database r220. Plasmids and insertion elements (ISs) were detected using MOB-suite v.3.1.9 [52], while prophages and CRISPR/Cas systems were detected using PHASTEST v.3.0 [53] and CRISPRCasFinder v.2.0.3 [54], respectively. Gene prediction and annotation were performed using Prokka v.1.14.6 [55]. Functional annotation was also performed using eggnog-mapper v.2.1.12 [56] and BlastKOALA v.3.1 [57]. Genes encoding bacteriocins were identified using BAGEL4 [58]. Virulence factors were detected using PanViTa v.1.1.8 [59] modified to use Virulence Factor Database (VFDB) full dataset [60]. The presence of bacterial toxins was verified using BlastKOALA.

Antimicrobial resistance genes were detected using Resistance Gene Identifier (RGI) v.6.0.3 from the Comprehensive Antibiotic Resistance Database (CARD) [61], PanViTa using CARD database, ResFinder v.4.7.2 [62] and BlastKOALA.

The ability to degrade HMOs was assessed in silico by predicting GHs and transporter proteins involved in their metabolism [63, 64]. Glycoside transporters and other proteins involved in the metabolism of HMO were identified using the Proteome Comparison Tool of BV-BRC (Shukla et al., 2025) by comparing the proteome of the four strains to the 78 proteins listed as part of the subsystem “HMO utilization” in Table 4 from Arzamasov et al., (2025) (Arzamasov et al., 2025). The reference proteins from *B. longum* SG596 used by Arzamasov et al., (2025) are not currently available on BV-BRC (genome ID 1679.217), so we also used Proteome Comparison Tool to map the original genome annotation from the JGI database (genome ID IMG_2513237171) to the current version of this genome on BV-BRC (genome ID 1679.478). We considered as orthologs proteins that had bidirectional hits an and an sequence identity > 60%.

### Cross-feeding Abilities Assessment

The cross-feeding capacity was evaluated by comparing the growth of selected probiotic candidates in monoculture or in combination with *Eubacterium callanderi* A1 (ECA1), a butyrate producer isolated in this study. Pre-processed bacterial cultures were either mono-cultured or co-cultured with ECA1 in a 1:1 (v/v) ratio in 50 mL of mMRS medium. Growth was monitored by recording the OD_600_ over 24 h at 37 °C under anaerobic conditions. Upon reaching the stationary phase, 1.5 mL of the bacterial culture was centrifuged at 10 000 x g for 5 min. The resulting pellet was used to quantify bacteria by qPCR, while the supernatant was utilized to evaluate SCFA production as described above.

The bacterial composition in mono- and co-cultures was quantified using quantitative PCR (qPCR). DNA was extracted with the Wizard^®^ Genomic DNA Purification Kit (Promega, Madison, WI, USA) following the manufacturer’s instructions and its concentration was measured via spectrophotometry (Nanodrop, Thermo Fisher, USA). DNA was diluted to 50 ng/µL and added to Takyon™ Rox SYBR^®^ MasterMix dTTP Blue (Eurogentec, Seraing, Belgium) according to the provider’s recommendations. The 16 S rRNA gene of the bacterial strains was amplified using the StepOne™ Real-Time PCR System (Thermo Fisher, USA) with the following primers:

- BLA1: F: 5’-ACACGGGAGTTAGGCCACC-3’, R: 5’-GGCGGAGTCGCTAGTAATCG-3’
- LRB1: F: 5’-GCTCCCTAGAAGGGTTACGC-3’, R: 5’-ACCGCGAGGTCAAGCTAATC-3’
- BBD1: F: 5’-CGAAGTGCCTTGCTCCCTAA-3’, R: 5’-ACTGAGATACGGCCCAGACT-3’
- BLE1: F: 5’-AGTCTGGGCCGTATCTCAGT-3’, R: 5’-AAAGCTTTCGCGGTATGGGA-3’
- ECA1: F: 5’-GCTGAGTCCTTGCGGTTCT-3’, R: 5’-TCGCCTGCATGAAGTTGGAG-3’

## Results

### Sample Collection, Bacteria Isolation and Growth Abilities

Given the well-established role of *Bifidobacterium* and *Lacticaseibacillus* species in promoting infant gastrointestinal health [2, 65, 66] we specifically targeted these groups by isolating colonies grown on MRS medium from stool samples collected at 1, 3, or 5 months ages. We selectively isolated 230 bacterial strains from MRS medium. Among them, 96 were classified under the genera of interest by 16 S sequencing. To ensure diversity and prevent duplication, we selected a maximum of one strain of each species per sample per month. This approach led to select 8 *Bifidobacterium* and 3 *Lacticaseibacillus* strains, *Bifidobacterium longum subsp. longum* A1 (BLA1), *Bifidobacterium breve* B1 (BBB1), *Bifidobacterium dentium* B1 (BDB1), *Bifidobacterium breve* C1 (BBC1), *Bifidobacterium breve* D1 (BBD1), *Bifidobacterium longum subsp. longum* E1 (BLE1), *Bifidobacterium pseudocatenulatum* E1 (BPE1), *Bifidobacterium breve* F1 (BBF1), *Lacticaseibacillus rhamnosus B1* (LRB1), *Lacticaseibacillus paracasei F1* (LPF1) and *Lacticaseibacillus rhamnosus A3* (LRA3) for in-vitro characterization. Detailed information on sampling time points and sample origin is provided in Supplementary Data 2.

**Fig. 1:**
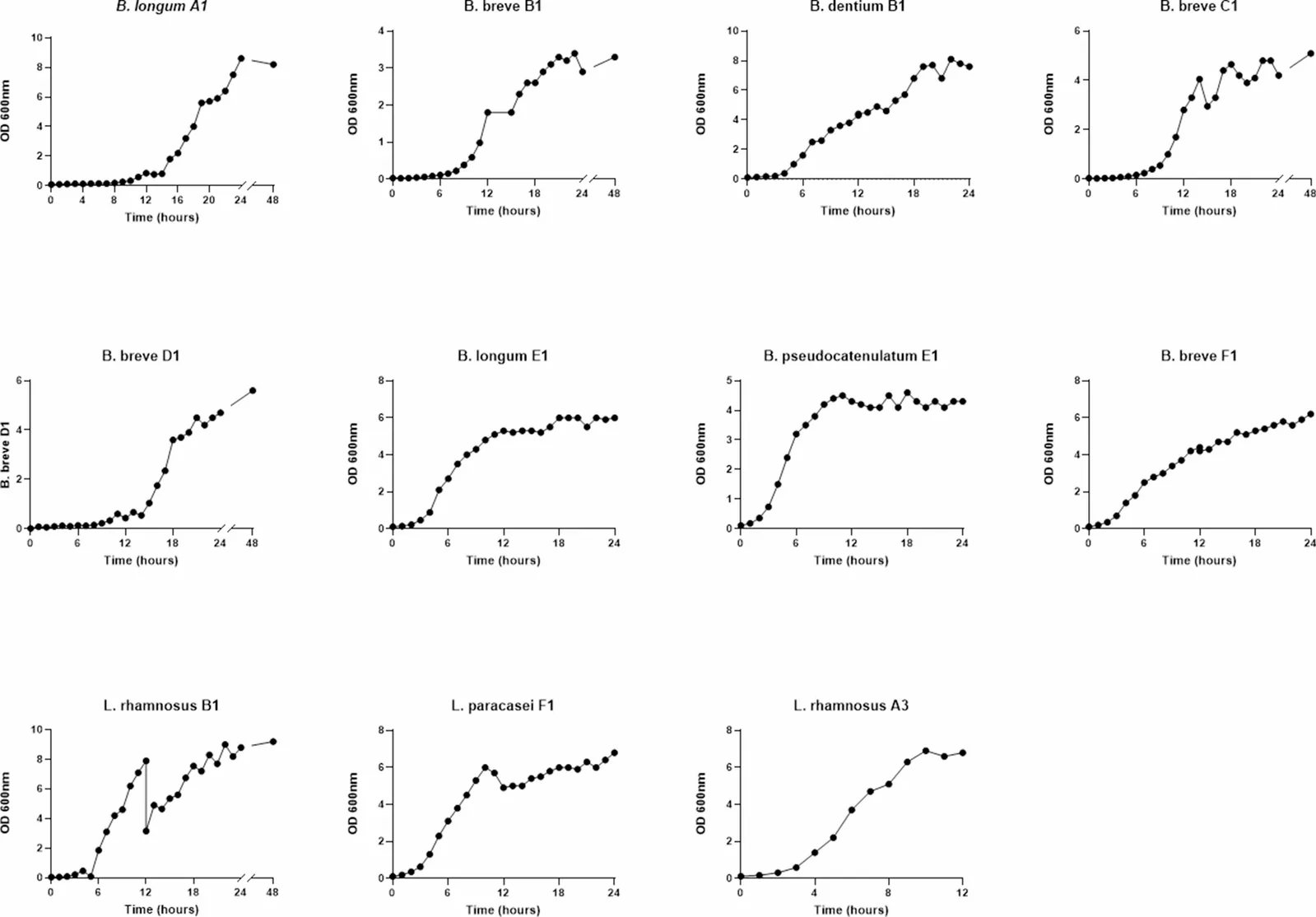
Growth curves of the 8 *Bifidobacterium* and 3 *Lacticaseibacillus* strains determined by OD_600_ monitoring over time. The assay was performed in MRS medium at 37 °C in anaerobic conditions

Growth of the strains were determined by monitoring OD_600_ over time (Fig. 1). Growth patterns varied among strains, reaching all stationary phase between 12 and 24 h of culture and a maximum OD_600_ between 4 and 10 after 24 h of incubation at 37 °C under anaerobic conditions.

### Metabolic profile

**Fig. 2:**
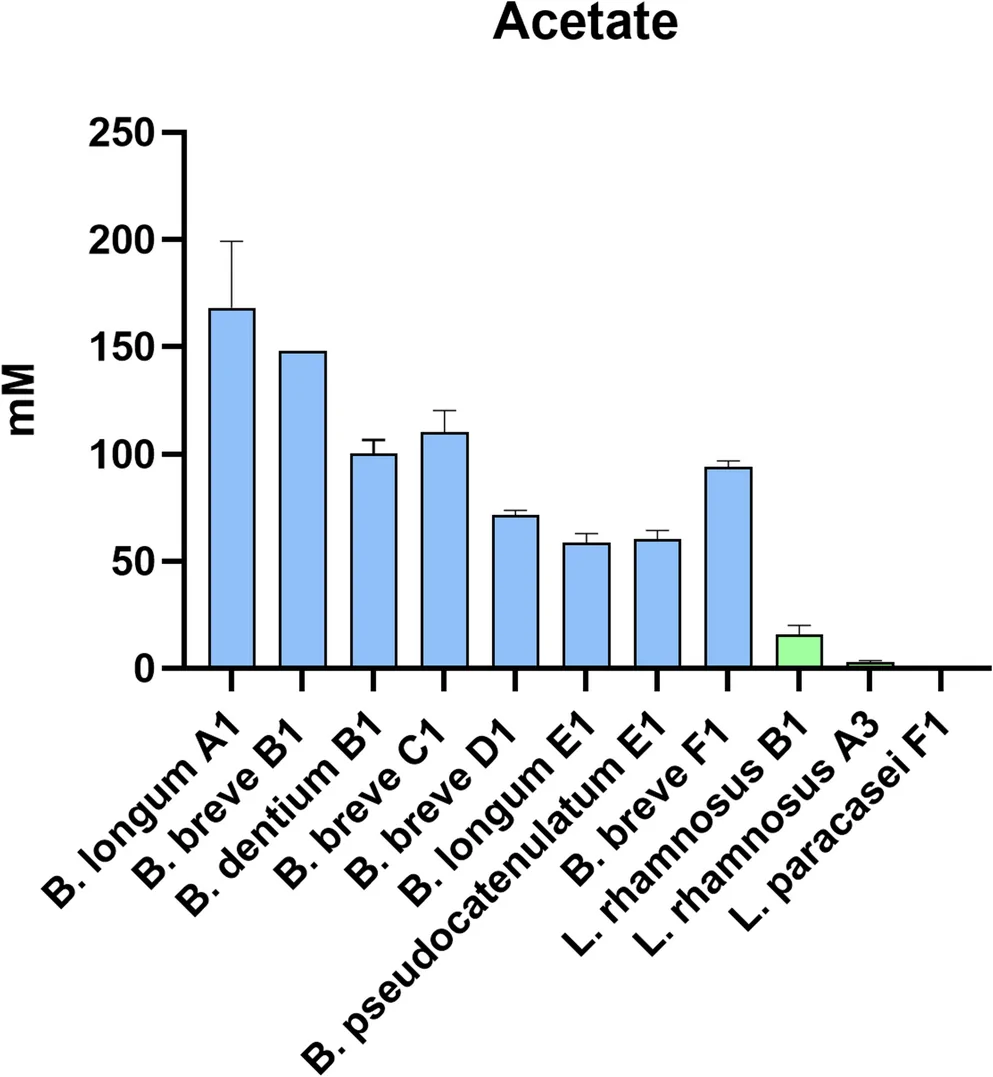
Acetate quantification of supernatant from stationary phase cultures of the *Bifidobacterium* species (blue) and *Lacticaseibacillus* species (green) using GC-FID. The initial values of the MRS medium were subtracted from the sample values. Data are expresses in mean ± SEM (*n* = 2)

Short-chain fatty acids (SCFAs) are key metabolites resulting from fermentation by gut microbiota, reflecting metabolic activity of the strains. To further characterize the 11 strains, SCFAs were quantified in the supernatant of stationary phase cultures (Fig. 2). *Bifidobacterium* species exhibit high production of acetate, particularly *B. longum* A1, which produced over 150 mM. No butyrate, propionate, or succinate levels were detected (data not shown). Conversely, no significant production of short-chain fatty acids (SCFA) was observed for the *Lacticaseibacillus s*trains.

API 50 galleries were utilized to evaluate carbohydrate fermentation of the 11 selected strains. All *Bifidobacterium* and *Lacticaseibacillus* strains utilized D-glucose, galactose, esculin, maltose, and lactose (Table 1). Additionally, all *Lacticaseibacillus* strains fermented ribose, D-fructose, D-mannose, mannitol, sorbitol, N-acetyl-glucosamine, amygdalin, arbutin, salicin, cellobiose, trehalose, melezitose, gentiobiose, and tagatose. LRA3 and LRB1 also fermented sorbose and rhamnose. Among the *Bifidobacterium* genus, the utilization of listed carbohydrates varied across all strains, although a similar pattern was observed for BLA1 and BLE1 strains.

**Table 1:** In vitro carbohydrates fermentation of the 11 probiotic candidates determined with API 50 galleries. Coloured indicator was used to determine the utilisation of each carbohydrate. Results were shown as “+++” for yellow, “++” for light green, “+” for dark green wells, indicating varying levels of carbohydrate utilization. A “-” symbol indicated no colour change and thus no carbohydrate utilization

	Bifidobacteria								Lacticaseibacilli		
	Species 1		Species 2				Species 3	Species 4	Species 1		Species 2
Carbohydrate	BLA1	BLE1	BBB1	BBC1	BBD1	BBF1	BDB1	BPE1	LRA3	LRB1	LPF1
Ctrl	-	-	-	-	-	-	-	-	-	-	-
Glycerol	-	-	-	-	-	-	-	-	-	-	-
Erythritol	-	-	-	-	-	-	-	-	-	-	-
D-Arabinose	-	-	-	-	-	-	-	-	-	-	-
L-Arabinose	+++	+++	-	-	-	-	+++	-	+	-	-
Ribose	-	-	+++	-	-	+++	+++	+++	+++	+++	+++
D-Xylose	+++	+++	-	-	-	-	+++	-	-	-	-
L-Xylose	-	-	-	-	-	-	-	-	-	-	-
Adonithol	-	-	-	-	-	-	-	-	-	-	-
Methyl xyloside	-	-	-	-	-	-	-	-	-	-	-
Galactose	+++	+++	+++	+++	+++	+++	++	+++	+++	+++	+++
D-Glucose	+++	+++	+++	+++	+++	+++	+++	+++	+++	+++	+++
D-Fructose	+++	+++	-	+++	-	+++	++	-	+++	+++	+++
D-Mannose	-	-	-	++	-	++	++	-	+++	+++	+++
Sorbose	-	-	-	-	-	-	-	-	+	+++	-
Rhamnose	-	-	-	-	-	-	-	-	+++	+++	-
Dulcitol	-	-	-	-	-	-	-	-	-	-	-
Insositol	-	-	-	-	-	-	-	-	-	-	-
Mannitol	+++	-	-	-	+++	+++	-	-	+++	+++	+++
Sorbitol	+++	-	+++	-	+++	+++	-	+++	+++	+++	+++
Methyl-D-mannoside	-	-	-	-	-	-	-	-	-	-	-
Methyl-D-glucoside	-	-	-	-	-	-	++	-	+++	+++	++
N-acetyl-glucosamine	-	-	-	-	-	+++	-	-	+++	+++	+++
Amygdaline	-	-	-	-	-	-	+++	+++	+++	+++	+++
Arbutine	-	-	-	-	-	+++	++	-	+++	+++	+++
Esculine	+++	+++	+++	+++	+++	+++	+++	+++	+++	+++	+++
Salicine	-	-	-	-	-	+++	+++	+	+++	+++	+++
Cellobiose	-	+++	-	-	-	-	++	-	+++	+++	+++
Maltose	+++	+++	+++	+++	+++	+++	+++	+++	+++	++	+++
Lactose	+++	+++	+++	+++	+++	+++	+	+	+++	+++	+++
Melibiose	+++	+++	++	+++	-	+++	-	+++	-	-	+++
Sucrose	+++	+++	-	-	+++	+++	++	+++	++	-	-
Trehalose	-	-	-	-	-	+++	-	+++	+++	+++	+++
Inuline	-	-	-	-	-	-	-	-	-	-	+++
Melezitose	+++	+++	-	-	-	+++	-	-	+++	+++	+++
Raffinose	+++	+++	+++	+++	+++	+++	-	+++	-	-	+++
Starch	-	++	-	-	-	++	+++	++	-	-	-
Glycogen	-	-	+++	+++	-	+++	+++	+++	-	-	-
Xylitol	-	-	-	-	-	++	-	-	-	-	-
Gentiobiose	-	-	-	-	-	-	+	+++	+++	++	++
Turanose	-	++	+++	+	++	++	-	+++	-	+++	+++
Lyxose	-	-	-	-	-	-	-	-	-	-	-
Tagatose	-	-	-	-	-	-	-	-	+++	+++	+++
D-Fucose	-	-	-	-	-	-	-	-	-	-	-
L-Fucose	-	-	++	-	-	++	-	-	-	-	-
D-Arabitol	-	-	-	-	-	-	-	-	-	-	-
L-Arabitol	-	-	-	-	-	-	-	-	-	-	-
Gluconate	-	-	-	-	-	-	-	-	-	++	-
2, Keto-gluconate	-	-	-	-	-	-	+	-	-	-	-
5, Keto-gluconate	-	-	-	-	-	-	-	-	-	-	-

#### Resistance to bile salts and low pH

Given the importance of probiotic viability during passage through gastro-intestinal tract, we assessed the survival capacities of the probiotic candidates in gastrointestinal tract conditions. This evaluation involved the evaluation of their resistance to bile salts and low pH levels.

**Fig. 3:**
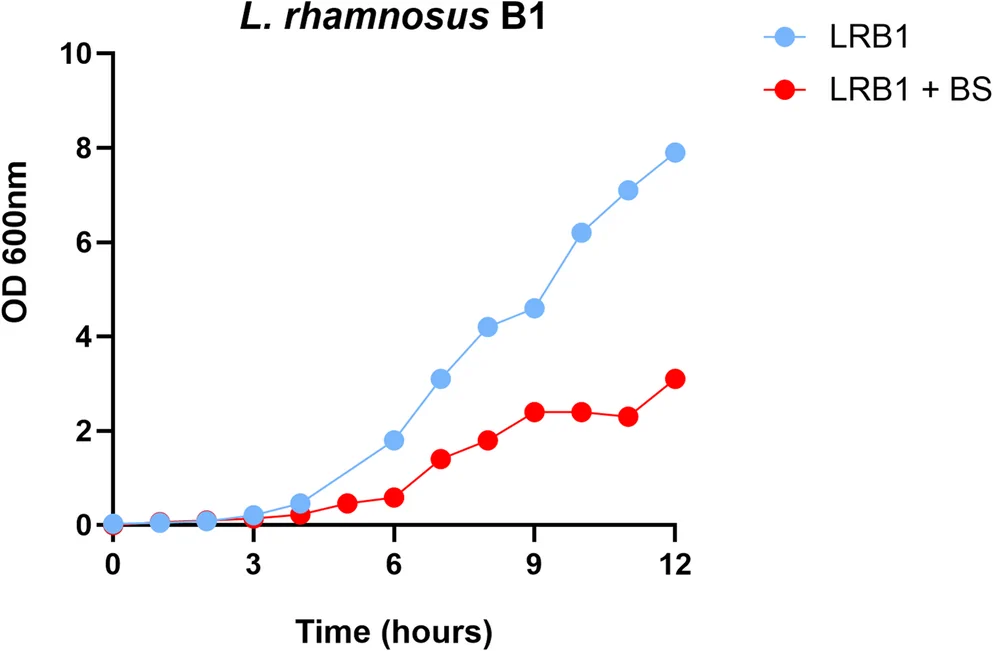
Effect of bile salts on the growth of strain LRB1. Growth was monitored by measuring optical density at 600 nm every hour in MRS medium supplemented with 0,3% of bile salts (red) or without bile salts (blue)

Firstly, growth curves of the strains were conducted in the presence of bile salts over 24 h to assess their ability to thrive under these conditions. The results revealed that out of the 11 strains tested, only LRB1 demonstrated growth in the presence of 0.3% bile salt, albeit not reaching the same levels observed in the absence of bile salts (Fig. 3).

**Fig. 4:**
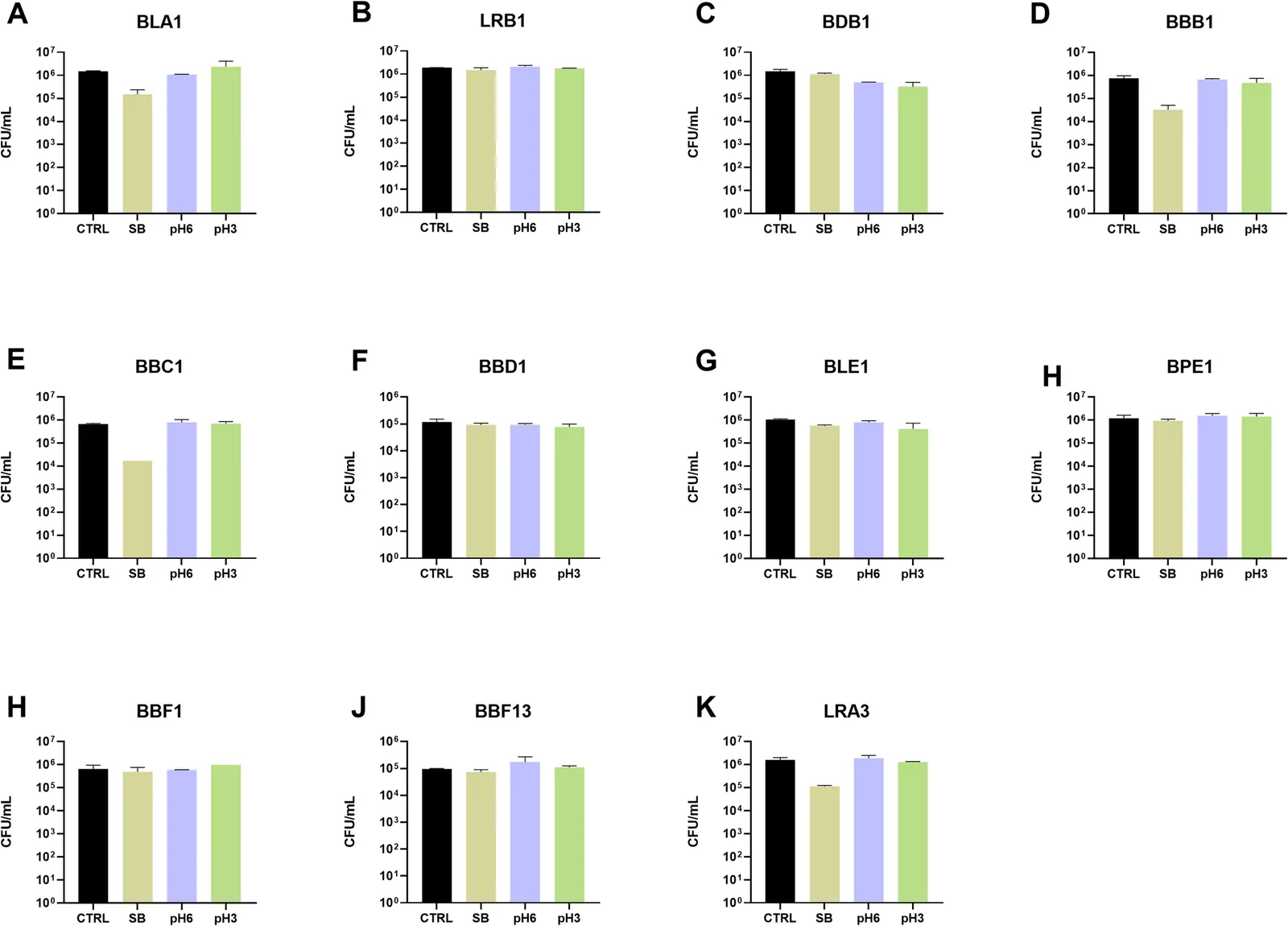
Counting results of the bacterial suspension after 1 h exposition to, PBS (black),0,3% bile salts (yellow), pH 3 (blue) and pH 6 (green). Data are expressed as mean ± SEM (*n* = 2)

Regarding bile salt shocks results, the strains BLA1, BBB1, BBC1 and LRA3 were sensitive to a 1-hour exposure to 0.3% bile salts, resulting in a two-log reduction in viable cell count compared to the control condition (Fig. 4A, D, E and K). In contrast, the remaining strains showed no difference compared to the control condition (Fig. 4B, C, F, G, H, I and J), demonstrating their ability to survive in these conditions. The same lack of sensitivity was observed under acidic conditions for all the tested strains.

### Minimum Inhibitory Concentration (MIC) determination

To ensure safety of the 11 strains, phenotypic antimicrobial susceptibility was investigated in-vitro using E-test assay according to EFSA recommendations. MIC values were determined as the lowest concentration inhibiting strain growth, with susceptibility defined by EFSA cut-off values. Among *Bifidobacterium* species, six strains (BLA1, BDB1, BBB1, BBD1, BBC1, and BLE1) showed sensitivity against the 8 antibiotics tested, since all the measured MIC were less than or equal to the cut-off values determined by EFSA (Table 2A). Both *B. pseudocatenulatum* and *B. breve* F1 exhibited resistance to gentamicin, with MIC values ranging between 64 and 256 mg/L. Furthermore, *B. breve* F1 demonstrated resistance to streptomycin, as evidenced by the absence of growth inhibition around the E-strip. Regarding *Lacticaseibacillus* species, LRB1, LRA3, and LPF1 were resistant to kanamycin and chloramphenicol (Table 2B). Additionally, LRA3 demonstrated resistance to gentamicin, and LPF1 showed resistance to clindamycin (Table 2B).

**Table 2:** Antimicrobial resistance of (A) *Bifidobacterium* strains (*n* = 2), (B) *L. rhamnosus* B1 and A3 (*n* = 4) and *L. paracasei* strains (*n* = 2). Minimum Inhibitory Concentration range values (MIC; mg/L) are compared to determined cut-off values, according to EFSA guidelines. Values highlighted in bold indicate resistance to the corresponding antibiotic with MIC higher than EFSA cut-off values. Abbreviations: AM, ampicillin; VA, vancomycin; GM, gentamycin; SM, streptomycin, EM, erythromycin; CM, clindamycin; CL, chloramphenicol and KM, kanamycin

AM	Bifidobacteriumcut-off values EFSA (mg/L)	*B. longum A1*	*B. breve* B1	*B. dentium* B1	*B. breve* C1	*B. breve* D1	*B. longum* E1	*B. pseudocatenulatum* E1	*B. breve* F1
	2	0.75–1	0.38–0.75	0.25–0.38	0.5–0.75	0.5–0.5	0.38–0.5	0.5	0.38–0.5
VA	2	0.75–0.75	1–1	0.75–1	0.75–1	1–1	0.5–0.75	0.38–0.5	1–1.5
GM	64	8–32	24–24	16–16	12–12	6–16	48–64	96–256	64–96
SM	128	8–48	24–24	24–48	12–16	12–24	64–32	128	No inhibition
EM	1	0.19–0.025	0.047–0.125	0.064–0.094	0.047–0.125	0.19–0.38	0.125–0.25	0.125–0.75	0.25
CM	1	0.064–0.094	0.5–0.75	0.023–0.032	0.25–0.38	0.25–0.38	0.064–0.094	0.064–0.125	0.064
TC	8	0.75–1	0.75–1	0.25–0.75	0.38–0.75	0.38–0.75	0.75	0.38–1.5	24
CL	4	0.75–1	0.75–1	1.5–1.5	1	0.75–0.75	1.5–3	2–1.5	1–1.5
AM	*Lacticaseibacillus rhamnosus* cut-off values EFSA (mg/L)	*L. rhamnosus* A3	*L. rhamnosus* B1	*Lacticaseibacillus paracasei* cut-off values EFSA (mg/L)	*L. paracasei* F1				
	4	0.75–1	0.75–1	4	0.75–1				
GM	16	8–24	6–12	32	8–12				
KM	64	192	128–192	64	128				
SM	32	32–96	16–96	64	48				
EM	1	1.5–6	0.38–1	1	0.5–1				
CM	4	0.75–1	1–3	4	6–8				
TC	8	2–6	0.75	4	1–1.5				
CL	4	12–16	6–12	4	6–8				

### Evaluation of Strains Probiotic Properties

**Fig. 5:**
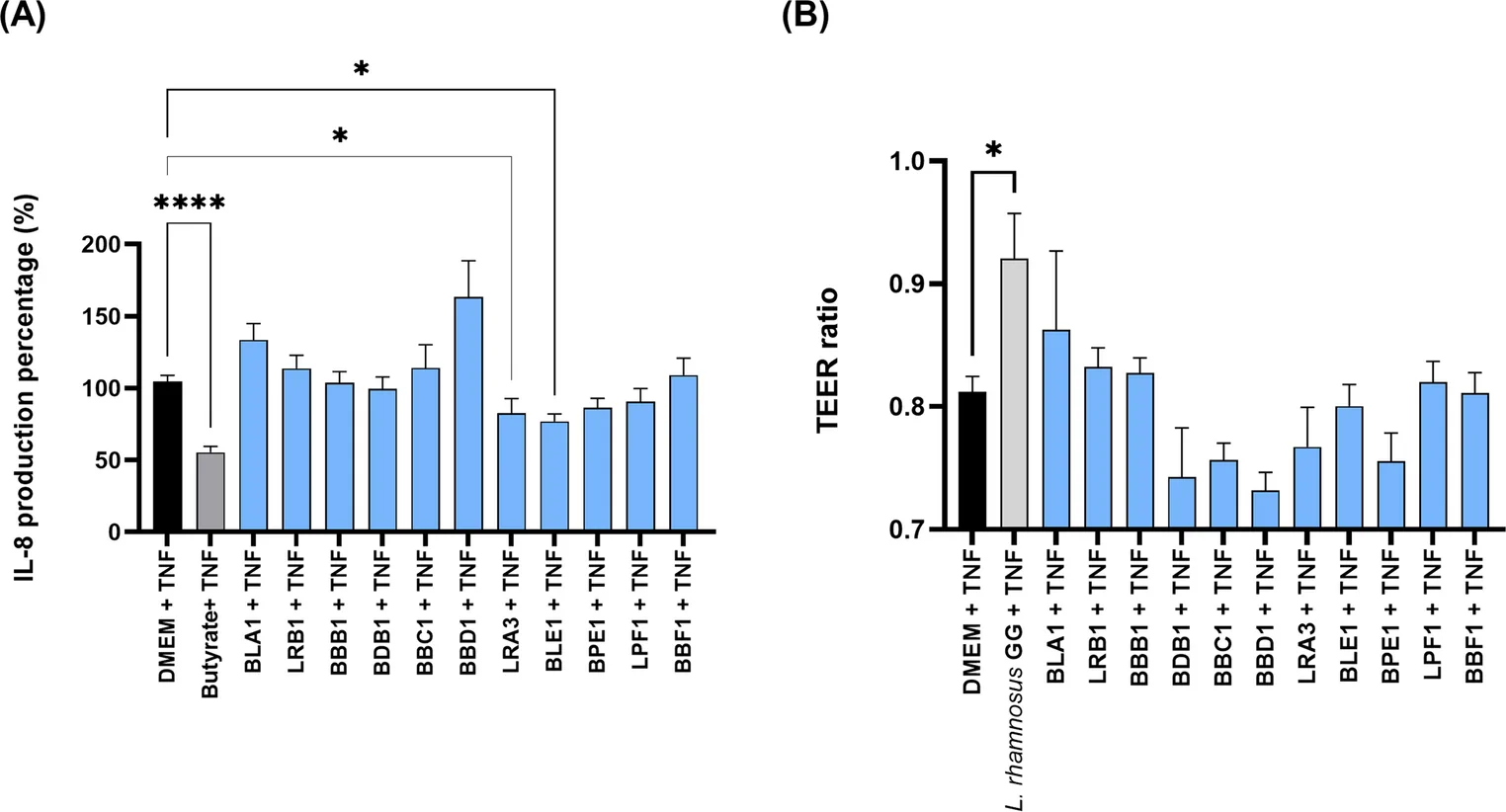
In vitro probiotic capacity assessment. Data are expressed as mean ± SEM (*n* = 3). **A** Il-8 production measurement after co-incubation of the 11 probiotics candidates with TNF-α stimulated HT-29 cells. Statistical significance was assessed using the Kruskal-Wallis test followed by the Dunn’s test, with significance levels identified as *p < 0.05, **p < 0.01, ***p < 0.001, or ****p < 0.0001. **B** Ratio of TEER signal recorded after co-incubation of the 11 strains with TNF-α stimulated Caco-2 cells. Statistical significance was assessed using Mann-Whitney test, with significance levels identified as *p < 0.05, **p < 0.01, ***p < 0.001, or ****p < 0.0001. Data are expressed as mean ± SEM (*n* = 3)

The 11 candidate probiotics strains were evaluated for their capacity to regulate the inflammatory response in the gut by quantifying IL-8 inhibition in TNF-alpha-stimulated HT-29 cells. Following 6 h of co-incubation, LRA3 and BLE1 demonstrated reductions in IL-8 production to 83% and 77%, respectively. However, their efficacy was slightly lower compared to the positive control, butyrate, which achieved a reduction to 55% (Fig. 5A).

Capacity of the strains to protect intestinal barrier was evaluated by measuring TEER signal in TNF-alpha-stimulated Caco-2 cells. After 24 h of incubation, none of the strains showed an increase in TEER signal compared to the reference strain *L. rhamnosus* GG (Fig. 5B).

Based on the results presented so far, an initial selection step was performed during the screening process. Several criteria were defined to maximize the probability of selecting the most promising probiotic candidates. To achieve a more diverse selection, we included at least one strain from each represented group (bifidobacteria and lactobacilli) in the selection. Safety was a key criterion, with antibiotic resistance phenotypes playing a key role in our selection. For this reason, among the *Bifidobacterium* strains that showed favourable antimicrobial susceptibility, BLA1 was chosen first for its consistently positive results across the tested parameters, particularly its ability to produce large amounts of acetate (Fig. 2). BLE1 was selected for its potential anti-inflammatory capabilities while BBD1 stood out for both good acetate production and bile salt resistance (Fig. 4). Since all three *Lacticaseibacillus* strains showed resistance to multiple antibiotics, additional tests were performed. The results were then compared to the antibiotic resistance profile of the LGG reference strain. LRB1 was selected for its lower resistance and the closest match to the LGG profile (Table 2).

Therefore, BLA1, BLE1, BBD1, and LRB1 were selected for further characterization.

### Whole Genome Analysis

Whole genome sequencing was performed for BLA1, BBD1, BLE1, and LRB1. The analysis aimed to detect potential Antimicrobial Resistance Genes (ARGs), virulence genes, plasmids, prophages, toxin production, biogenic amine formation, and bile salt hydrolases. Full results are provided in Supplementary Data 5. All genome assemblies were closed, with estimated completeness ranging from 99.91 to 99.99%, estimated contamination from 0.06 to 0.42%, and no chimeric contig detected. The genomes were classified as *B. breve* (BBD1) *B. longum subsp. longum* (BLA1 and BLE1) and *L. rhamnosus* (LRB1). Using VFDB and BlastKOALA, five virulence factors (*groEL2*, *rfbB*, *lisR*, *tufA* and *hasC*) and two hemolytic toxin genes (*tlyC* and *hlyIII*) were identified across all genomes. Additionally, two plasmids were found in BBLE1 genome, while complete prophage were identified in the genome of LRB1 and BLE1. Resistance gene analysis with PanViTa software (CARD) identified mupirocin (*ileS*), rifampicin (*rpoB*), and macrolide (*mefA*) resistance genes in the genomes of BLA1, BBD1, and BLE1. In the LRB1 genome, macrolide and beta-lactamase (*Isa*) resistance genes were found; however, none of these ARGs were located within mobile genetic elements (MGEs). A putative bile salt hydrolase gene (*cbh)* was detected in the BLA1, BBD1 and BLE1 genome. Genes associated with biogenic amine formation (*odcl*) were founded in the LRB1 genome and D-lactate dehydrogenase gene (*IdhA*) was identified in BLA1 strain.

### Human Milk Oligosaccharides (HMOs) Utilization

**Fig. 6:**
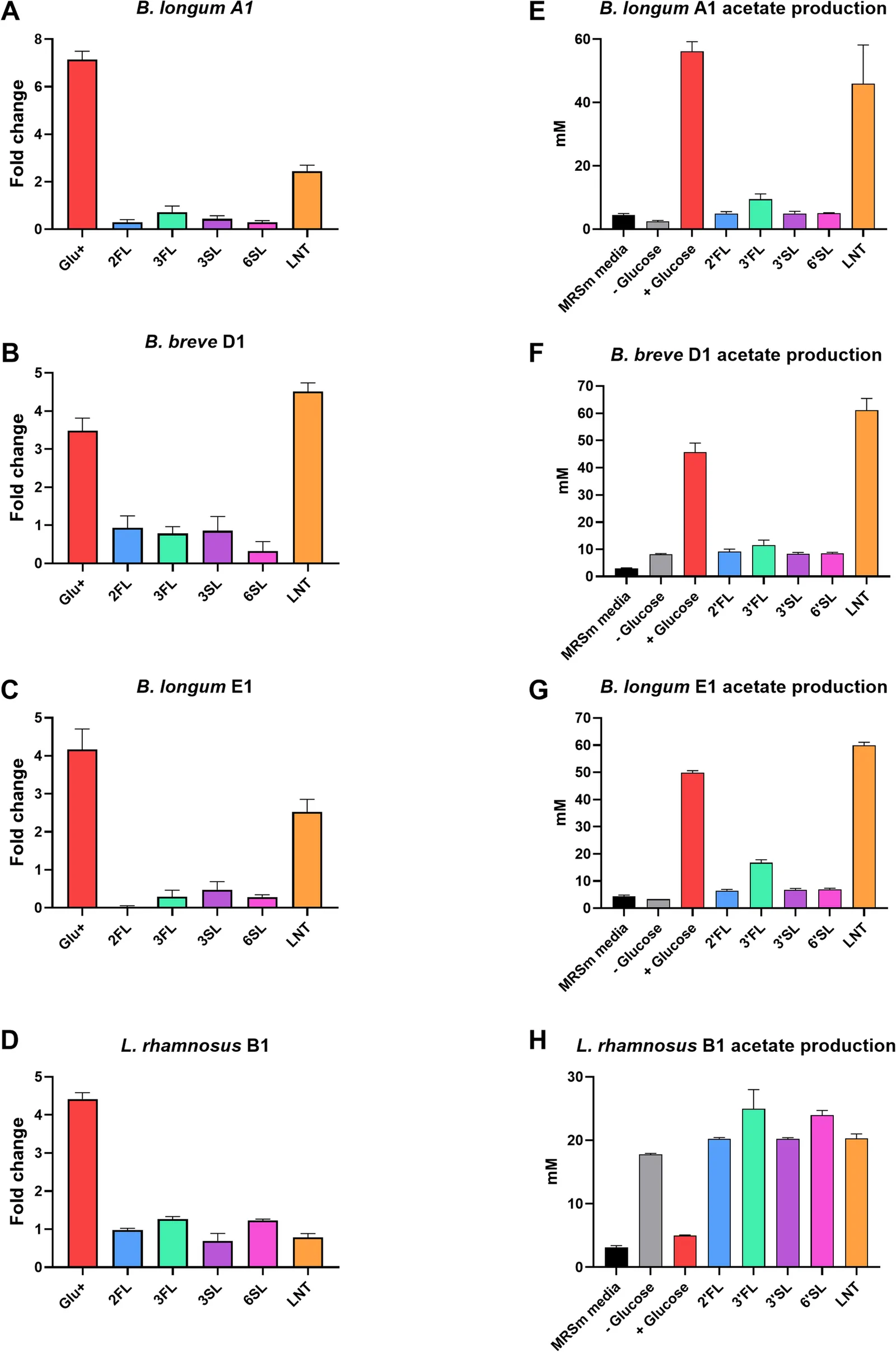
HMOs fermentation of the 4 selected strains assessed by (**A**-**D**) final growth expressed as OD_600_ fold change relative to the no-glucose control in the presence of glucose or individual HMOs, and (E-H) quantification of SCFA produced in the corresponding stationary-phase cultures. Growth was recorded using a Cerillo plate reader, which reports relative OD₆₀₀ values. The assay was carried out twice, with technical duplicate. Data are expressed in mean ± SEM (*n* = 4)

To assess the capacity of the four strains to degrade HMOs, their growth was examined in the presence of 2’FL, 3-FL, 3’-SL, 6’-SL, and LNT (2’-fucosyllactose, 3-fucosyllactose, 3’-sialyllactose, 6’-sialyllactose, and Lacto-N-tetraose), five HMOs commonly found in human breast milk [8, 9, 67]. Growth was quantified based on final OD₆₀₀ values expressed as fold change relative to the no-glucose control condition (Fig. 6A–D). Overall, *Bifidobacterium* species exhibited growth in the presence of LNT, although magnitude of the fold change varied among strains (Fig. 6). LNT supported the growth of BLA1 and BLE1 but to a lower extent than glucose (fold change of 2.44 vs 7.14 for BLA1 and 2.52 vs. 4.16 for BLE1, respectively; Fig. 6A and C). In contrast, strain BBD1 displayed higher fold change values when grown on LNT than on glucose (fold change of 4.52 vs. 3.48; Fig. 6B). Regarding the LRB1 strain, none of the HMOs were utilized (Fig. 6D).

To evaluate HMO degradation, the SCFA profiles of the strains were analysed with HMOs as the sole carbon source (Fig. 6). Only acetate was detected as a fermentation product in the *Bifidobacterium* strains. Similar acetate levels were observed across the different conditions in which growth occurred (Fig. 6E–G), ranging from approximately 40 to 60 mM. LRB1 produced comparable acetate levels in the absence of carbohydrate and in the presence of the five HMOs, under conditions where little to no growth was detected. In contrast, no acetate production was observed when LRB1 was grown on glucose, despite measurable growth under this condition (Fig. 6H).

The ability of candidate probiotics to degrade HMOs was evaluated by identifying glycoside hydrolase (GH) and transporters genes (Tables 3 and 4). These enzymes can break down HMOs by cleaving the complex glycosidic bonds between their monosaccharide units. Marked differences in GH repertoires were observed among the strains. Genes from GH2 and GH42 families, commonly associated with the degradation of HMOs, were detected in all four strains, although with variable copy numbers. Strain BBD1 carried 4 copies of GH2 genes, whereas 1 copy was identified in strains BLA1, LRB1, and BLE1. GHs associated with LNT degradation showed a more restricted distribution. GH20 genes were detected in strains BLA1, BBD1, and BLE1 but were absent from LRB1. In addition, GH112 genes, which are involved in lacto-N-biose metabolism, were detected in strains BLA1, BBD1, and BLE1.In contrast, GHs targeting fucosylated and sialylated HMOs were strain-specific. A GH29 gene was detected only in strain LRB1, while GH33 and GH95 genes were exclusively identified in strain BBD1, indicating a broader genetic potential for the utilization of fucosylated and sialylated HMOs in this strain. Finally, a GH136 gene was detected exclusively in strain LRB1.Comparative genomic analysis revealed differences in the repertoire of predicted carbohydrate transporters potentially associated with HMO utilization among the *Bifidobacterium* strains (Table 4). Homologs of the LNB/GNB transporter components GltA, GltB and GltC were detected in strain BLE1, whereas strains BLA1 and BBD1 encoded the LNT-associated transporter components GltA_LNT, GltB_LNT and GltC_LNT. In contrast, strain LRB1 lacked detectable homologs of these transport systems. Transporters predicted to be involved in the uptake of fucosylated HMOs were rare. Only strain BBD1 retained homologs of the FL2 permease components FL2_B and FL2_C, whereas no FL-type transporters were detected in strains BLA1, BLE1 or LRB1. A homolog of the LNnT-associated transporter NahS was detected only in strain BBD1. Homologs of NahC were identified in strains BLA1 and BLE1, whereas NahB was not detected in any of the genomes analyzed. Finally, the lactose/galactose transporter LacS was detected only in strain BLE1. These results highlight strain-specific differences in transporter repertoires, although the presence of homologous ABC transporter components does not necessarily imply identical substrate specificity.

**Table 3:**
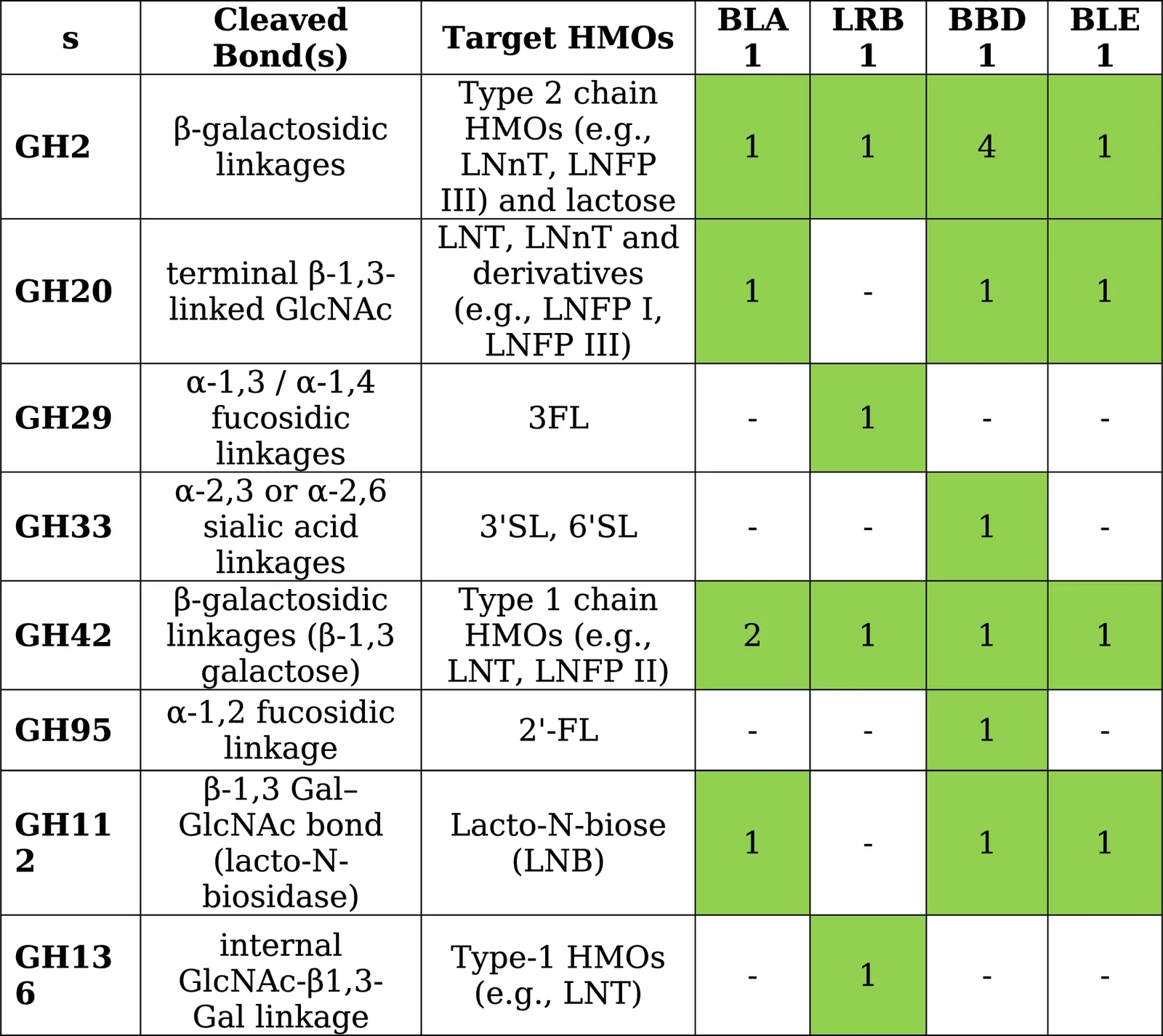
Number of detected GH enzyme genes (highlighted in green) in the genomes of BLA1, LRB1, BBD1, and BLE1, along with the corresponding glycosidic bond cleaved and the potentially targeted human milk oligosaccharides (HMOs)

**Table 4:** HMO transporter gene repertoire of the four probiotic candidates. Presence (+) or absence (−) of predicted HMO transporter genes identified by comparative genomic analysis

		BLA1	LRB1	BBD1	BLE1
LNB/LNT transporters	GltA	-	-	-	+
	GltB	-	-	-	+
	GltC	-	-	-	+
	GltF	-	-	-	-
	GltG	-	-	-	-
	GltH	-	-	-	-
	GltA_LNT	+	-	+	-
	GltB_LNT	+	-	+	-
	GltC_LNT	+	-	+	-
Fucosyl-HMO transporters (FL)	FL1_A	-	-	-	-
	FL2_A	-	-	-	-
	FL2_A2	-	-	-	-
	FL2_B	-	-	**+**	-
	FL2_C	-	-	**+**	-
	FL3_A	-	-	-	-
	FL3_B	-	-	-	-
	FL3_C	-	-	-	-
LNnT transporters	NahS	-	-	**+**	-
	NahB	-	-	-	-
	NahC	**+**	-	-	**+**
Broad-HMO transporters	HmoA	-	-	-	-
	HmoA2	-	-	-	-
	HmoA3	-	-	-	-
	HmoA4	-	-	-	-
	HmoA5	-	-	-	-
	HmoB	-	-	-	**+**
	HmoC	-	-	-	**+**
Galacto/lactose transporters	LacS	**+**	-	**+**	**+**

### Evaluation of Cross-feeding Capacity

**Fig. 7:**
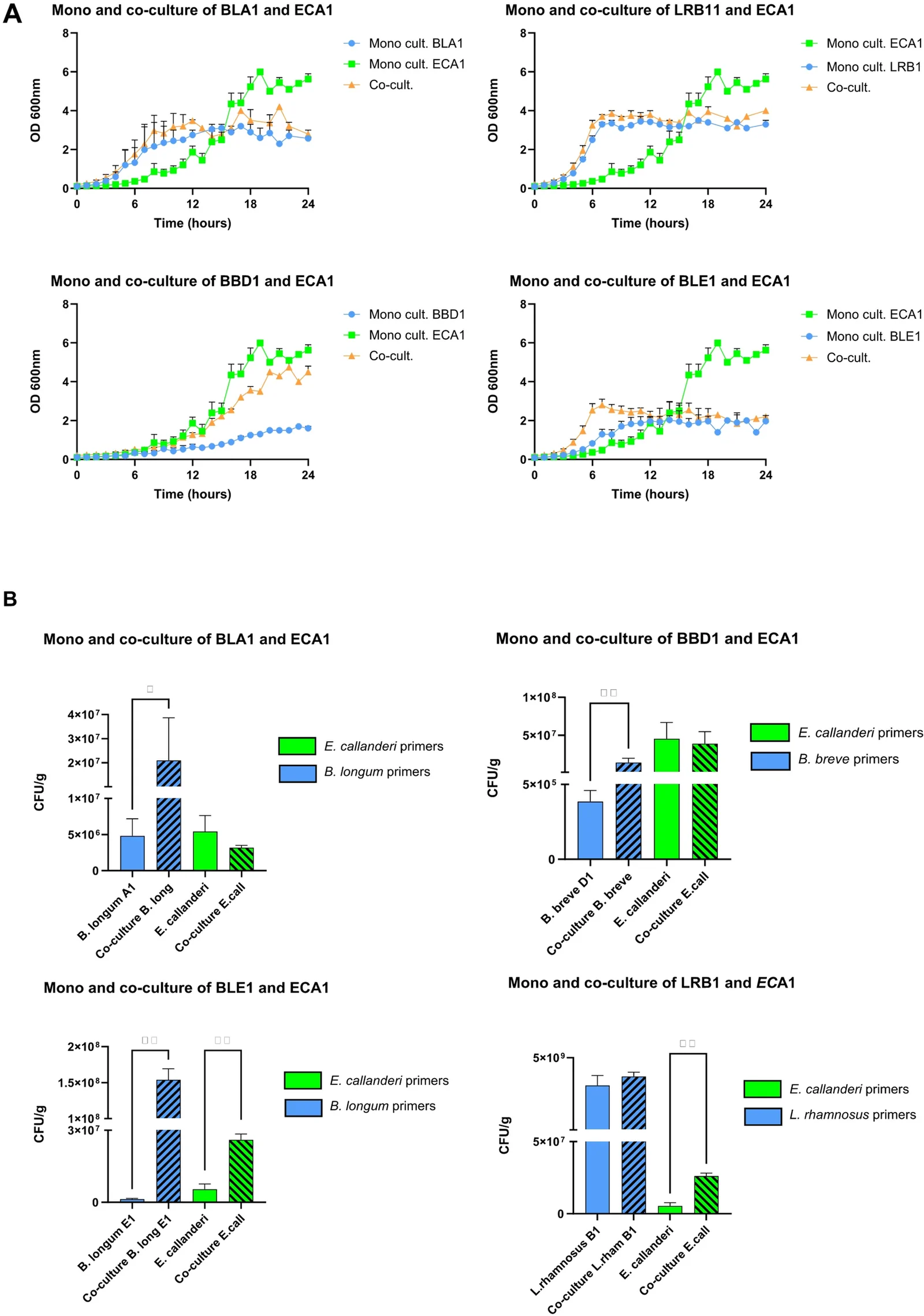
Growth profile determination of the 4 probiotic candidate and ECA1 in mono- and co- culture by (**A**) growth curve experiment and (**B**) qPCR quantification. Data are expressed as mean ± SEM (*n* = 2). Statistical significance between the monoculture and co-culture conditions was assessed using the Mann-Whitney U test. A p-value < 0.05 was considered statistically significant, and exact *p*-values are reported

**Fig. 8:**
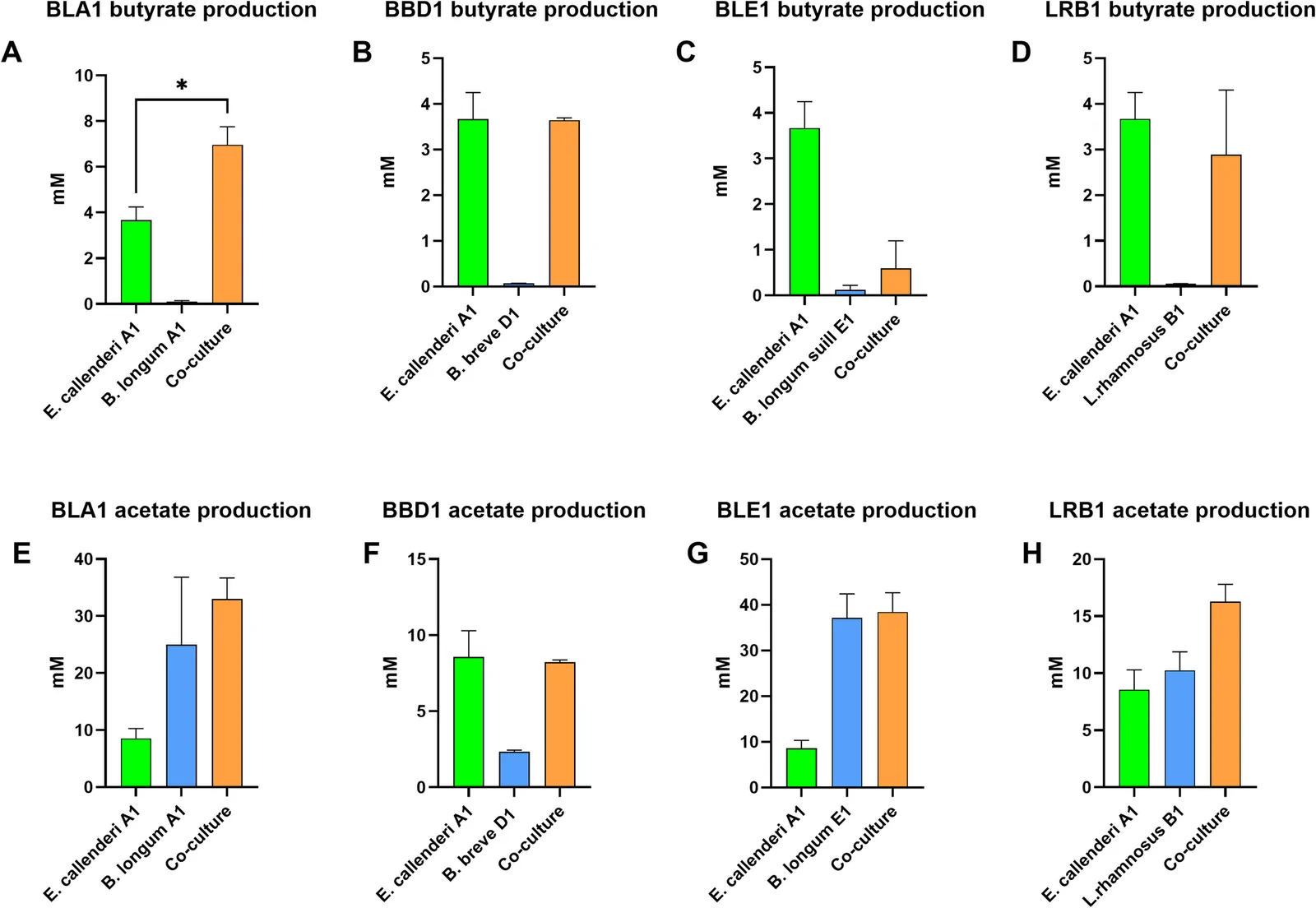
Quantification of SCFA levels in the supernatant for both mono- and co-cultures (in orange) of BLA1, BBD1, BLE1, LRB1 (in blue), and ECA1 (in green) after 24 h of growth using GC-FID. Data are expressed as mean ± SEM (*n* = 2). Statistical significance between each monoculture and the co-culture conditions was assessed using the Mann–Whitney U test. A *p*-value < 0.05 was considered statistically significant, and exact p-values are reported

To examine potential of our strains to cross-feed with butyrate producer bacteria present in the early life, we monitored their growth when cultured alone or with the butyrate producer *Eubacterium callanderi* A1 (ECA1) isolated in our study. The goal was to assess whether the probiotic strains could enhance ECA1’s biomass and/or metabolite production. As shown by both the growth-curve data and qPCR enumeration (Fig. 7A and B), none of the probiotic strains initially increased ECA1’s growth rate in co-culture compared to ECA1 alone. Interestingly, SCFA quantification revealed that the presence of BLA1 in the co-culture significantly boosted butyrate production by ECA1 (Fig. 8A) which cannot be explained by addition of individual butyrate production. In terms of acetate production, the amount generated was either comparable to the production of the probiotic strain in mono-culture or the cumulative acetate quantities produced by the two strains separately (Fig. 8E, F, G and H).

## Discussion

In infants, establishing a balanced gut microbiota is crucial for healthy development. A well-regulated microbiota supports digestive system maturation, regulates metabolism, and strengthens the immune system, reducing the risk of infections, asthma, and various inflammatory and metabolic disorders [13, 14, 68–70]. Beneficial bacteria, such as lactobacilli and bifidobacteria help limit systemic inflammation and provide protection against pathogens and respiratory infections [23, 69, 71, 72]. Their presence has also been associated with a lower incidence of infant colic, a reduced risk of allergies, and healthier weight development in infants [14, 70, 73]. Given these benefits, probiotic formulations containing strains from these bacterial groups have gained attention as a promising strategy to enhance gut microbiota balance and promote overall infant health [74–78].

Within this framework, the present study aimed to isolate and functionally characterize novel probiotic candidates specifically adapted to the ecological and nutritional constraints of early life, using strains isolated from the fecal microbiota of healthy, exclusively breastfed infants. Our objective was to identify strains with traits relevant to the perinatal window, encompassing birth, the first months of life, and the transition toward weaning. To this end, an in vitro screening was conducted on 11 isolates from healthy, exclusively breastfed infants including *B. longum* A1 and E1, *B. breve* B1, C1, D1 and F1, *B. dentium* B1, *B. pseudocatenulatum* E1, *L. rhamnosus* B1 and A3 and *L. paracasei* F1.

Selecting a probiotic requires evaluating safety, survival under host-associated condition to host conditions and functional efficacy. The safety of probiotic strains is a key criterion for selection, as antibiotic resistance genes represent a potential risk of horizontal transfer [79, 80]. Among the tested *Bifidobacterium* strains, BLA1, BDB1, BBB1, BBD1, BBC1, and BLE1 were sensitive to all antibiotics tested. However, BBPE1 and BBF13 showed resistance to gentamicin, and BBF13 was also resistant to streptomycin. While bifidobacteria are generally considered intrinsically sensitive to aminoglycosides [81, 82], rare cases of resistance to gentamicin and streptomycin have been reported [83]. Among *Lacticaseibacillus* species, all strains were resistant to chloramphenicol and kanamycin. LRA3 also resisted to gentamicin and erythromycin, LPF1 to clindamycin, and LRB1 to streptomycin. Lactobacilli are known for intrinsic resistance to aminoglycosides due to low membrane permeability and lack of active transport [81]. However, resistance to chloramphenicol, erythromycin, and clindamycin, antibiotics to which *Lacticaseibacillus* is typically sensitive, was unexpected [84] and may suggest the acquisition or activation of non-intrinsic resistance mechanisms [85]. To further assess safety, DNase activity was tested, as its presence could degrade host DNA or disrupt microbiota balance. Encouragingly, none of the 11 strains exhibited active DNase production, reinforcing their safety status.

A key property of probiotics is their ability to survive in the infant gut, particularly by resisting to bile salts and acidic pH [86, 87]. Probiotics that withstand these conditions are more likely to persist and exert beneficial effects. Among the tested strains, only LRB1 grew in the presence of bile salts, suggesting potential bile salt hydrolase (BSH) activity [88]. Interestingly, genome analysis detected putative BSH activity in BLA1 but not in LRB1. This apparent discrepancy highlights the complexity of linking genotype to phenotype and may reflect strain-specific regulation, differential gene expression, or the presence of divergent or unannotated BSH homologues [89, 90].

Beyond survival, probiotics exert their beneficial effects through functional activities that shape the intestinal ecosystem. Among these functions, metabolic outputs such as SCFA production is crucial for regulating intestinal pH, strengthening the epithelial barrier, and modulating immunity [91, 92]. As expected, *Lacticaseibacillus* strains produced no measurable SCFAs, while all *Bifidobacterium* strains generated significant amounts of acetate, with *B. longum* A1 reaching up to 200 mM. Acetate is known to contribute to protection against infections and to support mucosal integrity, both of which are essential for infant health [93].

In addition to metabolic activity, direct interactions with host immune and epithelial cells represent another important functional dimension of probiotic strains in early life. Given the close interaction between the early gut microbiota and the developing immune system, assessing bacterial strains for IL-8 modulation and epithelial barrier support provides insight into their potential to influence host immune responses [15, 44, 94, 95]. In this context, BLE1 and LRA3 reduced IL-8 production in HT-29 cells, consistent with the known anti-inflammatory properties of certain *Lacticaseibacillus* and *Bifidobacterium* species [35, 96, 97]. However, none of the strains enhanced the TEER signal in Caco-2 cells, suggesting no effect on tight junction protein expression [98, 99].

To identify the most promising probiotic candidate, a multi-criteria selection process was carried out. To broaden the spectrum of selected strains, at least one representative from each genus detected across all samples was included, provided the strain showed a positive result. Safety was considered by carefully assessing antibiotic resistance profiles, a key aspect of the screening process. Among the *Bifidobacterium* strains, BLA1 emerged as a leading candidate due to its particularly high acetate production (Fig. 2). BLE1 stood out for its potential to modulate inflammation (Fig. 7), while BBD1 was notable for its ability to resist bile salts (Fig. 5). Regarding *Lacticaseibacillus* strains, LRB1 was selected for its low level of antibiotic resistance and its strong similarity to the LGG strain antimicrobial profile (data not shown).

Whole Genome Sequencing (WGS) was conducted on the four selected strains to assess their safety. As expected, no virulence genes were detected. Resistance genes for macrolides (*mef*), tetracycline (*tetO*), beta-lactams (*penP*), rifampicin (*rpoB*), and lincosamides (*lsa*) were identified but were not linked to mobile genetic elements (MGEs) like plasmids or prophages, suggesting chromosomal integration and a low risk of horizontal transfer [100, 101]. These genes were also not expressed (Table 2), further supporting strain safety. The mupirocin resistance gene (*ileS*), found in *Bifidobacterium* strains, is considered as an intrinsic trait linked to a mutation in *ileS* (isoleucyl-tRNA synthetase), as reported by Serafini et al. (2011) [102]. According to the EFSA, intrinsic resistance does not pose a significant risk for food applications. In silico analysis using BlastKOALA, detected putative hemolytic toxin genes in BLA1, BLE1, BBD1, and LRB1, but in vitro tests confirmed they were inactive. Additionally, two plasmids were identified in BBLE1 and a complete prophage in LRB1 and BLE1, which are common features in lactic acid bacteria that could contribute to their genomic plasticity and adaptation [103–105]. Genes associated with biogenic amine and D-lactate production were found in LRB1 and BLA1 respectively, warranting further functional analysis to confirm safety. Overall, the genomic analysis of these probiotic strains aligns with existing literature on *Bifidobacterium* and *Lacticaseibacillus*, both of which are traditionally regarded as safe for probiotic use.

Beyond safety considerations, the functional integration of probiotic strains within early-life microbial networks represents another critical aspect of their relevance for perinatal applications. Cross-feeding is a fundamental trophic mechanism, particularly active during neonatal primary colonization of the gut. Recent studies have highlighted the contribution of early metabolic interactions in shaping and stabilizing the gut microbiota, especially between lactate- and acetate-producing bacteria (such as *Lactobacillus* and *Bifidobacterium*) and butyrate-producing bacteria (BPB), like *Anaerostipes caccae* and several *Eubacterium* species [16, 18, 72, 86–88]. In infants, BPBs are detected at low abundance during the pre-weaning period, with a marked increase occurring around weaning, concomitant with dietary diversification [18, 92, 106–109]. This transition is functionally significant, as butyrate plays a central role in epithelial energy metabolism, immune regulation, and barrier integrity in the mature gut [14, 66]. The gradual increase of BPBs and butyrate production may play a central role in the functional maturation of the intestine and in host-microbiota communication [106, 110, 111]. Accordingly, the perinatal period represents a window during which probiotic strategies may aim to support the metabolic networks that facilitate the later establishment of butyrogenic functions, rather than to introduce BPBs prematurely.

To explore this hypothesis, we investigated the interactions between the four selected strains and *Eubacterium callanderi* A1 (ECA1), isolated from the faeces of children participating in the study and capable of producing butyrate from lactate and acetate [111–114]. Although *E. callanderi* is not typically described as a dominant member of the pre-weaning gut microbiota, its use in this study was primarily motivated by its functional butyrogenic capacity and its relevance to cross-feeding mechanisms described for early-life BPBs, such as *Anaerobutyricum hallii* [115]. In this experimental context, ECA1 was used as a functional representative to assess the feasibility of cross-feeding interactions rather than to reflect the typical taxonomic composition of the infant gut microbiota. Notably, only BLA1 significantly stimulated butyrate production by ECA1, though without notable growth enhancement. These results suggest that BLA1 releases lactate and acetate in balanced proportions, creating a metabolically favourable environment for ECA1’s butyrate activity, as previously reported [115, 116].BLA1’s ability to cross-feed with ECA1 highlights its probiotic potential to promote balanced development of the intestinal microbiota in newborns.

From a nutritional ecology perspective, the ability to utilize milk-derived substrates such as HMOs represents a key functional trait for probiotic candidates intended for early-life applications [7]. Assessing HMO metabolism therefore provides insight into the ecological fitness of strains within the breastfed infant gut. Although prebiotics such as galacto-oligosaccharides (GOS) and fructo-oligosaccharides (FOS) are commonly used to fortify infant formulae, they were not used as sole carbohydrate sources or positive growth controls in this study, as the objective was to specifically assess adaptation to the breast milk-driven nutritional landscape characteristic of early life rather than general oligosaccharide fermentability. In this context, glucose was used as a positive control to ensure robust and comparable growth across all strains in the semi-defined medium. At the phenotypic level, all selected *Bifidobacterium* strains produced acetate when grown on LNT as the sole carbon source, supporting their capacity to metabolize this abundant neutral HMO. This finding is consistent with the well-established role of bifidobacteria as primary HMO consumers in the infant gut and reinforces their relevance as probiotic candidates for early-life applications [8, 117, 118]. In contrast, LRB1 did not grow on HMOs, in agreement with previous reports showing that most lactobacilli lack the enzymatic machinery required for HMO degradation [118]. These observations highlight the distinct ecological roles of bifidobacteria and lactobacilli within the infant gut ecosystem, where bifidobacteria typically function as primary degraders of HMOs.

Genomic analyses largely supported these observations. Members of the GH2 and GH42 families were detected across all strains, whereas GH20 enzymes involved in LNT degradation were restricted to the bifidobacterial strains. These enzymes are known to participate in the intracellular degradation pathway of type I HMOs, where LNT is sequentially processed after transport into the cell [119]. In contrast, GH112 enzymes participate in lacto-N-biose metabolism rather than in the direct degradation of LNT itself, as previously described for bifidobacterial LNB/GNB pathways [120]. In addition, strain BBD1 harbored GHs targeting fucosylated and sialylated HMOs, suggesting a broader genetic potential for HMO utilization. Fucosylated and sialylated HMOs represent structurally diverse substrates that can support niche specialization among infant-associated bifidobacterial [121]. However, this broader enzymatic repertoire was not fully reflected under the in vitro conditions tested, which may indicate regulatory constraints, substrate accessibility limitations, or the requirement for specific transporter systems.

Comparative genomic analysis further revealed strain-specific differences in the repertoire of transporter homologs potentially associated with HMO uptake. Strains BLA1 and BBD1 encoded homologs of the LNT-associated transporter components GltA_LNT, GltB_LNT and GltC_LNT, whereas strain BLE1 carried homologs of the GltA, GltB and GltC components of the LNB/GNB transporter family. In addition, BBD1 retained homologs of the FL2 permease components associated with the transport of fucosylated HMOs, whereas these systems were not detected in the other strains after applying stricter filtering criteria. A homolog of the LNnT-associated transporter NahS was detected only in strain BBD1, while NahC homologs were identified in strains BLA1 and BLE1. In contrast, strain LRB1 lacked most transporter homologs typically associated with HMO uptake. Given the high sequence similarity among carbohydrate ABC transporters in bifidobacteria, the presence of homologous transporter components should be interpreted with caution, as substrate specificity cannot always be inferred solely from sequence similarity [121]. Nevertheless, variability in transporter repertoires may influence how efficiently individual strains access milk-derived glycans and therefore affect their ecological competitiveness in the breastfed infant gut*.*

Interestingly, LRB1 produced acetate under HMO-containing conditions at levels comparable to those observed in the carbohydrate-free control, despite the absence of detectable growth. This acetate production is therefore unlikely to reflect HMO metabolism and more plausibly corresponds to basal or maintenance metabolism, as previously described for lactic acid bacteria under carbon-limited conditions [122]. This observation underscores the limited relevance of lactobacilli as direct HMO utilizers, while acknowledging that their metabolic activity may still contribute indirectly to the early gut ecosystem, for instance through metabolic cross-feeding interactions with HMO-degrading bifidobacteria.

Taken together, these results indicate that the selected *Bifidobacterium* strains possess strain-dependent but functionally relevant capacities to utilize HMOs, supporting their suitability for perinatal probiotic applications. Efficient utilization of dominant HMOs such as LNT is widely considered an important ecological trait enabling bifidobacteria to establish and persist in the breastfed infant gut. While HMO utilization alone does not capture the full complexity of infant gut colonization, it represents a meaningful indicator of adaptation to the breastfed infant niche. Future work should explore these metabolic traits in more complex nutritional contexts, including mixed-feeding scenarios and interactions with other early-life microbial functions.

While this study provides a comprehensive in vitro characterization of probiotic candidates isolated from the early-life gut microbiota, several limitations should be acknowledged. First, the functional assays were conducted under controlled in vitro conditions, which do not fully recapitulate the complexity of the infant gut environment, including host-microbe interactions, microbial community dynamics, and evolving dietary inputs. In addition, the number of strains selected for in-depth characterization reflects a targeted screening strategy rather than an exhaustive exploration of early-life microbial diversity. Importantly, the intended application of the probiotic candidates identified in this study is focused on the perinatal and early infancy period, encompassing birth, the first months of life, and the transition towards weaning, rather than later infancy or toddlerhood. This developmental window represents a critical phase during which the gut microbiota is highly dynamic and functionally immature, and during which metabolic cross-feeding interactions may contribute to the progressive establishment of key microbial functions, including butyrate production. In this context, the aim is not to directly supplement butyrate-producing bacteria, which typically expand after weaning, but rather to support the early microbial networks that facilitate their later establishment.

Future studies should aim to validate these findings in more complex experimental systems, including multi-species consortia representative of early-life microbial networks, mixed-feeding nutritional conditions, and ex vivo or in vivo models better reflecting host-microbe interactions. In particular, evaluating selected strains, such as BLA1, in synbiotic formulations combining probiotics with specific HMOs represents a logical next step to assess their functional performance in a more physiologically relevant context. Overall, this study provides a rational and scalable framework for the identification of probiotic candidates adapted to the perinatal and early infancy periods, with the long-term objective of supporting microbiota maturation and intestinal homeostasis during this critical developmental window.

In conclusion, this study provides a comprehensive and integrated in vitro characterization of probiotic candidates isolated from the early-life gut microbiota, combining phenotypic, functional, and genomic approaches. Among the strains evaluated, *Bifidobacterium longum* A1 emerged as a particularly robust candidate, displaying a favourable safety profile, strong acetate production, the capacity to utilize LNT, and the ability to engage in metabolically relevant cross-feeding interactions with a butyrate-producing strain. These properties are especially relevant within the perinatal context, where the gut microbiota is highly dynamic and shaped by milk-derived substrates and early metabolic cooperation. While several strains exhibited individual probiotic traits of interest, BLA1 combined multiple functional characteristics aligned with the nutritional and ecological constraints of early life, supporting its prioritization for further preclinical investigation.

Overall, this work contributes to the rational selection of probiotic candidates tailored to the perinatal and early infancy periods and provides a methodological framework for linking microbial function, ecological fitness, and probiotic potential during the earliest stages of gut microbiota development.

## Supplementary Information

Below is the link to the electronic supplementary material.

Supplementary Material 1 (DOCX 27.4 KB)

## Data Availability

The complete genome sequences generated and analyzed during this study are part of an industrial research project and are subject to confidentiality agreements. These data may be made available upon reasonable request to the corresponding author, pending approval from the industrial partner and in accordance with applicable data sharing policies.
