# Supplementary material for: Isolation and Characterization of Bifidobacterium longum A1, an Isolate from Faeces of an Exclusively Breast-fed Infant with Probiotic Potential
Source: Probiotics Antimicrob Proteins. 2026 Apr 14;18(6):8105–28. doi: 10.1007/s12602-026-11017-7 (PMC13518436; doi:10.1007/s12602-026-11017-7)
Supplement: Supplementary file 1 [file 12602_2026_11017_MOESM1_ESM.docx]

**Supplementary data**

Vaginal birth

Full-term birth (≥ 37 weeks of gestation)

Exclusively breastfed

No intrapartum antibiotic administration

No maternal antibiotic use during the third trimester

No maternal probiotic use during the third trimester

No chronic medical conditions in the mother

No chronic medications taken by the mother

Maternal BMI < 25 at the start of pregnancy

No gestational diabetes

No diagnosed pathologies in the newborn

No medical treatment for the newborn (except standard vitamin supplementation)

No parental opposition to participation

Optional (but recommended): not the first child

Optional (but recommended): mother lives in a rural area and has pets

Supplementary data 1- Eligibility criteria for newborns participating in the study.

| Isolates strains name | Infant | Month of life |
| --- | --- | --- |
| *B. longum* A1 | A | 1 |
| *B. breve* B1 | B | 1 |
| *B. dentium* D1 | B | 1 |
| *L. rhamnosus* B1 | B | 1 |
| *B. breve* C1 | C | 1 |
| *B. breve* D1 | D | 1 |
| *B. longum* E1 | E | 1 |
| *B. pseudocatenulatum* E1 | E | 1 |
| *B. breve* F1 | F | 1 |
| *L. paracasei* F1 | F | 1 |
| *L. rhamnosus* A3 | A | 3 |

Supplementary data 2 - Origin of the bacterial strains isolated from infants and age at sampling

| **Isolated strains name** | **V1-V9 16S rRNA gene sequence** |
| --- | --- |
| BLA1 | ACACGGGAGTTAGGCCACCGGCTTCGGGTGCTGCCCACTTTCATGACTTGACGGGCGGTGTGTACAAGGCCCGGGAACGCATTCACCGCGACGTTGCTGATTCGCGATTACTAGCGACTCCGCCTTCACGCAGTCGAGTTGCAGACTGCGATCCGAACTGAGACCGGTTTTCAGGGATCCGCTCCGCGTCGCCGCGTCGCATCCCGTTGTACCGGCCATTGTAGCATGCGTGAAGCCCTGGACGTAAGGGGCATGATGATCTGACGTCATCCCCACCTTCCTCCGAGTTAACCCCGGCGGTCCCCCGTGAGTTCCCGGCATAATCCGCTGGCAACACGGGGCGAGGGTTGCGCTCGTTGCGGGACTTAACCCAACATCTCACGACACGAGCTGACGACGACCATGCACCACCTGTGAACCCGCCCCGAAGGGAAGCCGTATCTCTACGACCGTCGGGAACATGTCAAGCCCAGGTAAGGTTCTTCGCGTTGCATCGAATTAATCCGCATGCTCCGCCGCTTGTGCGGGCCCCCGTCAATTTCTTTGAGTTTTAGCCTTGCGGCCGTACTCCCCAGGCGGGATGCTTAACGCGTTAGCTCCGACACGGAACCCGTGGAACGGGCCCCACATCCAGCATCCACCGTTTACGGCGTGGACTACCAGGGTATCTAATCCTGTTCGCTCCCCACGCTTTCGCTCCTCAGCGTCAGTAACGGCCCAGAGACCTGCCTTCGCCATTGGTGTTCTTCCCGATATCTACACATTCCACCGTTACACCGGGAATTCCAGTCTCCCCTACCGCACTCAAGCCCGCCCGTACCCGGCGCGGATCCACCGTTAAGCGATGGACTTTCACACCGGACGCGACGAACCGCCTACGAGCCCTTTACGCCCAATAATTCCGGATAACGCTTGCACCCTACGTATTACCGCGGCTGCTGGCACGTAGTTAGCCGGTGCTTATTCAACGGGTAAACTCACTCTCGCTTGCTCCCCGATAAAAGAGGTTTACAACCCGAAGGCCTCCATCCCTCACGCGGCGTCGCTGCATCAGGCTTGCGCCCATTGTGCAATATTCCCCACTGCTGCCTCCCGTAGGAGTCTGGGCCGTATCTCAGTCCCAATGTGGCCGGTCGCCCTCTCAGGCCGGCTACCCGTCGAAGCCACGGTGGGCCGTTACCCCGCCGTCAAGCTGATAGGACGCGACCCCATCCCATACCGCGAAAGCTTTCCCAGAAGACCATGCGATCAACTGGAGCATCCGGCATTACCACCCGTTTCCAGGAGCTATTCCGGTGTATGGGGCAGGTCGGTCACGCATTACTCACCCGTTCGCCACTCTCACCACCAAGCAAAGCCTGATGTGATCCCGTTCGACTGTCCTACG |
| BBB1 | TAGTCCCTCCCGCACGGAGTTAGGCCACCGGCTTCGGGTGCTGCCCACTTTCATGACTTGACGGGCGGTGTGTACAAGGCCCGGGAACGCATTCACCGCGACGTTGCTGATTCGCGATTACTAGCGACTCCGCCTTCACGCAGTCGAGTTGCAGACTGCGATCCGAACTGAGACCGGTTTTCAGGGATCCGCTCCAGCTCGCGCTGTCGCATCCCGTTGTACCGGCCATTGTAGCATGCGTGAAGCCCTGGACGTAAGGGGCATGATGATCTGACGTCATCCCCACCTTCCTCCGAGTTAACCCCGGCGGTCCCCCGTGAGTTCCCGGCACAATCCGCTGGCAACACGGGGCGAGGGTTGCGCTCGTTGCGGGACTTAACCCAACATCTCACGACACGAGCTGACGACGACCATGCACCACCTGTGAACCCGCCCCGAAGGGAAACCCCATCTCTGGGATCGTCGGGAACATGTCAAGCCCAGGTAAGGTTCTTCGCGTTGCATCGAATTAATCCGCATGCTCCGCCGCTTGTGCGGGCCCCCGTCAATTTCTTTGAGTTTTAGCCTTGCGGCCGTACTCCCCAGGCGGGATGCTTAACGCGTTAGCTCCGACACGGAACCCGTGGAACGGGCCCCACATCCAGCATCCACCGTTTACGGCGTGGACTACCAGGGTATCTAATCCTGTTCGCTCCCCACGCTTTCGCTCCTCAGCGTCAGTAACGGCCCAGAGACCTGCCTTCGCCATTGGTGTTCTTCCCGATATCTACACATTCCACCGTTACACCGGGAATTCCAGTCTCCCCTACCGCACTCAAGCCCGCCCGTACCCGGCGCGGATCCACCGTTAAGCGATGGACTTTCACACCGGACGCGACGAACCGCCTACGAGCCCTTTACGCCCAATAATTCCGGATAACGCTTGCACCCTACGTATTACCGCGGCTGCTGGCACGTAGTTAGCCGGTGCTTATTCGAAAGGTACACTCAACAAAAAATGCCTTGCTCCCTAACAAAAGAGGTTTACAACCCGAAGGCCTCCATCCCTCACGCGGCGTCGCTGCATCAGGCTTGCGCCCATTGTGCAATATTCCCCACTGCTGCCTCCCGTAGGAGTCTGGGCCGTATCTCAGTCCCAATGTGGCCGGTCGCCCTCTCAGGCCGGCTACCCGTCGAAGCCATGGTGGGCCGTTACCCCGCCATCAAGCTGATAGGACGCGACCCCATCCCATGCCGCAAAGGCTTTCCCAACACACCATGCGGTGTGATGGAGCATCCGGCATTACCACCCGTTTCCAGGAGCTATTCCGGTGCATGGGGCAGGTCGGTCACGCATTACTCACCCGTTCGCCACTCTCACCACCAGGCAAAGCCCGATGGATCCCGTTCGACTTTCCTACG |
| BDB1 | AGGGCTCCATCCCGCAAGGGGTTAGGCCACCGGCTTCGGGTGCTGCCCACTTTCATGACTTGACGGGCGGTGTGTACAAGGCCCGGGAACGCATTCACCGCGGCGTTGCTGATCCGCGATTACTAGCGACTCCGCCTTCATGGAGTCGGGTTGCAGACTCCAATCCGAACTGAGACCGGTTTTCAGGGATCCGCTCCATGTCGCCATGTCGCATCCCGCTGTACCGGCCATTGTAGCATGCGTGAAGCCCTGGACGTAAGGGGCATGATGATCTGACGTCATCCCCACCTTCCTCCGAGTTGACCCCGGCGGTCCCCCGTGAGTTCCCACCATGACGTGCTGGCAACACAGGGCGAGGGTTGCGCTCGTTGCGGGACTTAACCCAACATCTCACGACACGAGCTGACGACGACCATGCACCACCTGTGAACCCGCCCCGAAGGGAGGCCATATCTCTACGGCTGTCGGGAACATGTCAAGCCCAGGTAAGGTTCTTCGCGTTGCATCGAATTAATCCGCATGCTCCGCCGCTTGTGCGGGCCCCCGTCAATTTCTTTGAGTTTTAGCCTTGCGGCCGTACTCCCCAGGCGGGATGCTTAACGCGTTAGCTCCGACACGGAACCCGTGGAACGGGCCCCACATCCAGCATCCACCGTTTACGGCGTGGACTACCAGGGTATCTAATCCTGTTCGCTCCCCACGCTTTCGCTCCTCAGCGTCAGTGACGGCCCAGAGACCTGCCTTCGCCATTGGTGTTCTTCCCGATATCTACACATTCCACCGTTACACCGGGAATTCCAGTCTCCCCTACCGCACTCCAGCCCGCCCGTACCCGGCGCAGACCCACCGTTAAGCGATGGGCTTTCACACCGGACGCGACGAACCGCCTACGAGCCCTTTACGCCCAATAATTCCGGATAACGCTTGCACCCTACGTATTACCGCGGCTGCTGGCACGTAGTTAGCCGGTGCTTATTCGAAAGGTACACTCACCCCCGAAGGGCTTGCTCCCGATCAAAAGCGGTTTACAACCCGAAGGCCTCCATCCCGCACGCGGCGTCGCTGCATCAGGCTTGCGCCCATTGTGCAATATTCCCCACTGCTGCCTCCCGTAGGAGTCTGGGCCGTATCTCAGTCCCAATGTGGCCGGTCGCCCTCTCAGGCCGGCTACCCGTCGAAGCCATGGTGGGCCGTTACCCCGCCATCAAGCTGATAGGACGCGACCCCATCCCATACCGATGGAATCTTTCCCGGAAGGACATGCATCCAACCGGAGCATCCGGCATTACCACCCGTTTCCAGGAGCTATTCCGGTGTATGGGGCAGGTCGGTCACGCATTACTCACCCGTTCGCCACTCTCACCCGGGAGCGAACTCCCCGGGATCCCGTTCGACTGCATTATCC |
| BBC1 | ATCCATCAGGCTTTGCTTGGTGGTGAGAGTGGCGAACGGGTGAGTAATGCGTGACCGACCTGCCCCATGCACCGGAATAGCTCCTGGAAACGGGTGGTAATGCCGGATGCTCCATCACATCGCATGGTGTGTTGGGAAAGCCTTTGCGGCATGGGATGGGGTCGCGTCCTATCAGCTTGATGGCGGGGTAACGGCCCACCATGGCTTCGACGGGTAGCCGGCCTGAGAGGGCGACCGGCCACATTGGGACTGAGATACGGCCCAGACTCCTACGGGAGGCAGCAGTGGGGAATATTGCACAATGGGCGCAAGCCTGATGCAGCGACGCCGCGTGAGGGATGGAGGCCTTCGGGTTGTAAACCTCTTTTGTTAGGGAGCAAGGCACTTTGTGTTGAGTGTACCTTTCGAATAAGCACCGGCTAACTACGTGCCAGCAGCCGCGGTAATACGTAGGGTGCAAGCGTTATCCGGAATTATTGGGCGTAAAGGGCTCGTAGGCGGTTCGTCGCGTCCGGTGTGAAAGTCCATCGCTTAACGGTGGATCCGCGCCGGGTACGGGCGGGCTTGAGTGCGGTAGGGGAGACTGGAATTCCCGGTGTAACGGTGGAATGTGTAGATATCGGGAAGAACACCAATGGCGAAGGCAGGTCTCTGGGCCGTTACTGACGCTGAGGAGCGAAAGCGTGGGGAGCGAACAGGATTAGATACCCTGGTAGTCCACGCCGTAAACGGTGGATGCTGGATGTGGGGCCCGTTCCACGGGTTCCGTGTCGGAGCTAACGCGTTAAGCATCCCGCCTGGGGAGTACGGCCGCAAGGCTAAAACTCAAAGAAATTGACGGGGGCCCGCACAAGCGGCGGAGCATGCGGATTAATTCGATGCAACGCGAAGAACCTTACCTGGGCTTGACATGTTCCCGACGATCCCAGAGATGGGGTTTCCCTTCGGGGCGGGTTCACAGGTGGTGCATGGTCGTCGTCAGCTCGTGTCGTGAGATGTTGGGTTAAGTCCCGCAACGAGCGCAACCCTCGCCCCGTGTTGCCAGCGGATTATGCCGGGAACTCACGGGGGACCGCCGGGGTTAACTCGGAGGAAGGTGGGGATGACGTCAGATCATCATGCCCCTTACGTCCAGGGCTTCACGCATGCTACAATGGCCGGTACAACGGGATGCGACAGTGCGAGCTGGAGCGGATCCCTGAAAACCGGTCTCAGTTCGGATCGCAGTCTGCAACTCGACTGCGTGAAGGCGGAGTCGCTAGTAATCGCGAATCAGCAACGTCGCGGTGAATGCGTTCCCGGGCCTTGTACGCACCGCCCGTCAAGTCATGAAAGTGGGCAGCACCCGAAGCCGGTGGCCTATAGCT |
| BBD1 | GAGAGATTAGGCCACCGGCTTCGGGTGCTGCCCACTTTCATGACTTGACGGGCGGTGTGTACAAGGCCCGGGAACGCATTCACCGCGACGTTGCTGATTCGCGATTACTAGCGACTCCGCCTTCACGCAGTCGAGTTGCAGACTGCGATCCGAACTGAGACCGGTTTTCAGGGATCCGCTCCAGCTCGCGCTGTCGCATCCCGTTGTACCGGCCATTGTAGCATGCGTGAAGCCCTGGACGTAAGGGGCATGATGATCTGACGTCATCCCCACCTTCCTCCGAGTTAACCCCGGCGGTCCCCCGTGAGTTCCCGGCATAATCCGCTGGCAACACGGGGCGAGGGTTGCGCTCGTTGCGGGACTTAACCCAACATCTCACGACACGAGCTGACGACGACCATGCACCACCTGTGAACCCGCCCCGAAGGGAAACCCCATCTCTGGGATCGTCGGGAACATGTCAAGCCCAGGTAAGGTTCTTCGCGTTGCATCGAATTAATCCGCATGCTCCGCCGCTTGTGCGGGCCCCCGTCAATTTCTTTGAGTTTTAGCCTTGCGGCCGTACTCCCCAGGCGGGATGCTTAACGCGTTAGCTCCGACACGGAACCCGTGGAACGGGCCCCACATCCAGCATCCACCGTTTACGGCGTGGACTACCAGGGTATCTAATCCTGTTCGCTCCCCACGCTTTCGCTCCTCAGCGTCAGTAACGGCCCAGAGACCTGCCTTCGCCATTGGTGTTCTTCCCGATATCTACACATTCCACCGTTACACCGGGAATTCCAGTCTCCCCTACCGCACTCAAGCCCGCCCGTACCCGGCGCGGATCCACCGTTAAGCGATGGACTTTCACACCGGACGCGACGAACCGCCTACGAGCCCTTTACGCCCAATAATTCCGGATAACGCTTGCACCCTACGTATTACCGCGGCTGCTGGCACGTAGTTAGCCGGTGCTTATTCGAAAGGTACACTCAACACGAAGTGCCTTGCTCCCTAACAAAAGAGGTTTACAACCCGAAGGCCTCCATCCCTCACGCGGCGTCGCTGCATCAGGCTTGCGCCCATTGTGCAATATTCCCCACTGCTGCCTCCCGTAGGAGTCTGGGCCGTATCTCAGTCCCAATGTGGCCGGTCGCCCTCTCAGGCCGGCTACCCGTCGAAGCCATGGTGGGCCGTTACCCCGCCATCAAGCTGATAGGACGCGACCCCATCCCATGCCGCAAAGGCTTTCCCAACACACCATGCGGTGTGATGGAGCATCCGGCATTACCACCCGTTTCCAGGAGCTATTCCGGTGCATGGGGCAGGTCGGTCACGCATTACTCACCCGTTCGCCACTCTCACCACCAGGCAAAGCCCGATGGATCCCGTTCGACTTGCATTA |
| BLE1 | GGGCTCCATCCCACAAGGGGTTAGGCCACCGGCTTCGGGTGCTGCCCACTTTCATGACTTGACGGGCGGTGTGTACAAGGCCCGGGAACGCATTCACCGCGACGTTGCTGATTCGCGATTACTAGCGACTCCGCCTTCACGCAGTCGAGTTGCAGACTGCGATCCGAACTGAGACCGGTTTTCAGGGATCCGCTCCGCGTCGCCGCGTCGCATCCCGTTGTACCGGCCATTGTAGCATGCGTGAAGCCCTGGACGTAAGGGGCATGATGATCTGACGTCATCCCCACCTTCCTCCGAGTTAACCCCGGCGGTCCCCCGTGAGTTCCCGGCATAATCCGCTGGCAACACGGGGCGAGGGTTGCGCTCGTTGCGGGACTTAACCCAACATCTCACGACACGAGCTGACGACGACCATGCACCACCTGTGAACCCGCCCCGAAGGGAAGCCGTATCTCTACGACCGTCGGGAACATGTCAAGCCCAGGTAAGGTTCTTCGCGTTGCATCGAATTAATCCGCATGCTCCGCCGCTTGTGCGGGCCCCCGTCAATTTCTTTGAGTTTTAGCCTTGCGGCCGTACTCCCCAGGCGGGATGCTTAACGCGTTAGCTCCGACACGGAACCCGTGGAACGGGCCCCACATCCAGCATCCACCGTTTACGGCGTGGACTACCAGGGTATCTAATCCTGTTCGCTCCCCACGCTTTCGCTCCTCAGCGTCAGTAACGGCCCAGAGACCTGCCTTCGCCATTGGTGTTCTTCCCGATATCTACACATTCCACCGTTACACCGGGAATTCCAGTCTCCCCTACCGCACTCAAGCCCGCCCGTACCCGGCGCGGATCCACCGTTAAGCGATGGACTTTCACACCGGACGCGACGAACCGCCTACGAGCCCTTTACGCCCAATAATTCCGGATAACGCTTGCACCCTACGTATTACCGCGGCTGCTGGCACGTAGTTAGCCGGTGCTTATTCAACGGGTAAACTCACTCTCGCTTGCTCCCCGATAAAAGAGGTTTACAACCCGAAGGCCTCCATCCCTCACGCGGCGTCGCTGCATCAGGCTTGCGCCCATTGTGCAATATTCCCCACTGCTGCCTCCCGTAGGAGTCTGGGCCGTATCTCAGTCCCAATGTGGCCGGTCGCCCTCTCAGGCCGGCTACCCGTCGAAGCCACGGTGGGCCGTTACCCCGCCGTCAAGCTGATAGGACGCGACCCCATCCCATACCGCGAAAGCTTTCCCAGAAGACCATGCGATCAACTGGAGCATCCGGCATTACCACCCGTTTCCAGGAGCTATTCCGGTGTATGGGGCAGGTCGGTCACGCATTACTCACCCGTTCGCCACTCTCACCACCAAGCAAAGCCCGATGGATCCCGTTCGACTTGCATTACC |
| BPE1 | ATTAAGGCACACCGGCTTCGGGTGCTACCCACTTTCATGACTTGACGGGCGGTGTGTACAAGGCCCGGGAACGCATTCACCGCGGCGTTGCTGATCCGCGATTACTAGCGACTCCGCCTTCATGGAGTCGGGTTGCAGACTCCAATCCGAACTGAGACCGGTTTTCAGGGATCCGCTCCACGTCGCCGTGTCGCATCCCGTTGTACCGGCCATTGTAGCATGCGTGAAGCCCTGGACGTAAGGGGCATGATGATCTGACGTCATCCCCACCTTCCTCCGAGTTGACCCCGGCGGTCCCCCGTGAGTTCCCACCATGACGTGCTGGCAACACAGGGCGAGGGTTGCGCTCGTTGCGGGACTTAACCCAACATCTCACGACACGAGCTGACGACGACCATGCACCACCTGTGAACCCGCCCCGAAGGGAGGCCATATCTCTACGGCTGTCGGGAACATGTCAAGCCCAGGTAAGGTTCTTCGCGTTGCATCGAATTAATCCGCATGCTCCGCCGCTTGTGCGGGCCCCCGTCAATTTCTTTGAGTTTTAGCCTTGCGGCCGTACTCCCCAGGCGGGATGCTTAACGCGTTAGCTCCGACACGGAACCCGTGGAACGGGCCCCACATCCAGCATCCACCGTTTACGGCGTGGACTACCAGGGTATCTAATCCTGTTCGCTCCCCACGCTTTCGCTCCTCAGCGTCAGTAACGGCCCAGAGACCTGCCTTCGCCATTGGTGTTCTTCCCGATATCTACACATTCCACCGTTACACCGGGAATTCCAGTCTCCCCTACCGCACTCCAGCCCGCCCGTACCCGGCGCAGATCCACCGTTAAGCGATGGACTTTCACACCGGACGCGACGAACCGCCTACGAGCCCTTTACGCCCAATAATTCCGGATAACGCTTGCACCCTACGTATTACCGCGGCTGCTGGCACGTAGTTAGCCGGTGCTTATTCGAAAGGTACACTCACCCGAAGGCTTGCTCCCGATCAAAAGCGGTTTACAACCCGAAGGCCGTCATCCCGCACGCGGCGTCGCTGCATCAGGCTTGCGCCCATTGTGCAATATTCCCCACTGCTGCCTCCCGTAGGAGTCTGGGCCGTATTTCAGTCCCAATGTGGCCGGTCGCCCTCTCAGGCCGGCTACCCGTCGTAGGCTCGGTGGGCCGTTACCCCGCCGACTACCTGATAGGACGCGACCCCATCCCATACCGATGAAATCTTTCCCGACACCCCATGCGAGGAGTCGGAGCATCCGGCATTACCACCCGTTTCCAGGAGCTATTCCGGTGTATGGGGCAGGTCGGTCACGCATTACTCACCCGTTCGCCACTCTCACCACCAAGCAAAGCCTGATTGAT |
| BBF1 | AGACTACTACACCGGCTTCGGGTGCTGCCCACTTTCATGACTTGACGGGCGGTGTGTACAAGGCCCGGGAACGCATTCACCGCGACGTTGCTGATTCGCGATTACTAGCGACTCCGCCTTCACGCAGTCGAGTTGCAGACTGCGATCCGAACTGAGACCGGTTTTCAGGGATCCGCTCCAGCTCGCGCTGTCGCATCCCGTTGTACCGGCCATTGTAGCATGCGTGAAGCCCTGGACGTAAGGGGCATGATGATCTGACGTCATCCCCACCTTCCTCCGAGTTAACCCCGGCGGTCCCCCGTGAGTTCCCGGCACAATCCGCTGGCAACACGGGGCGAGGGTTGCGCTCGTTGCGGGACTTAACCCAACATCTCACGACACGAGCTGACGACGACCATGCACCACCTGTGAACCCGCCCCGAAGGGAAACCCCATCTCTGGGATCGTCGGGAACATGTCAAGCCCAGGTAAGGTTCTTCGCGTTGCATCGAATTAATCCGCATGCTCCGCCGCTTGTGCGGGCCCCCGTCAATTTCTTTGAGTTTTAGCCTTGCGGCCGTACTCCCCAGGCGGGATGCTTAACGCGTTAGCTCCGACACGGAACCCGTGGAACGGGCCCCACATCCAGCATCCACCGTTTACGGCGTGGACTACCAGGGTATCTAATCCTGTTCGCTCCCCACGCTTTCGCTCCTCAGCGTCAGTAACGGCCCAGAGACCTGCCTTCGCCATTGGTGTTCTTTCCCGATATCTACACATTTCCACCGTTACACCGGGAATTCCAGTCTCCCCTACCGCAATCAAAGCCCGCCCGTACCCGGCGCGGATCCACCGTTAAGCGATGGACTTTCACACCGGACGCGACGAACCGCCTACGAGCCCTTTACGCCCAATAATTCCGGATAACGCTTGCACCCTACGTATTACCGCGGCTGCTGGCACGTAGTTAGCCGGTGCTTATTCGAAAGGTACACTCAACACAAAGTGCCTTGCTCCCTAACAAAAGAGGTTTACAACCCGAAGGCCTCCATCCCTCACGCGGCGTCGCTGCATCAGGCTTGCGCCCATTGTGCAATATTCCCCACTGCTGCCTCCCGTAGGAGTCTGGGCCGTATCTCAGTCCCAATGTGGCCGGTCGCCCTCTCAGGCCGGCTACCCGTCGAAGCCATGGTGGGCCGTTACCCCGCCATCAAGCTGATAGGACGCGACCCCATCCCATGCCGCAAAGGCTTTCCCAACACACCATGCGGTGTGATGGAGCATCCGGCATTACCACCCGTTTCCAGGAGCTATTCCGGTGCATGGGGCAGGTCGGTCACGCATTACTCACCCGTTCGCCACTCTCACCACCAAGCAAAGTTACGACT |
| LRB1 | ACTTAAACGGCTCGCTCCCTAGAAGGGTTACGCCACCGGCTTCGGGTGTTACAAACTCTCATGGTGTGACGGGCGGTGTGTACAAGGCCCGGGAACGTATTCACCGCGGCGTGCTGATCCGCGATTACTAGCGATTCCGACTTCGTGTAGGCGAGTTGCAGCCTACAGTCCGAACTGAGAATGGCTTTAAGAGATTAGCTTGACCTCGCGGTCTCGCAACTCGTTGTACCATCCATTGTAGCACGTGTGTAGCCCAGGTCATAAGGGGCATGATGATTTGACGTCATCCCCACCTTCCTCCGGTTTGTCACCGGCAGTCTTACTAGAGTGCCCAACTAAATGCTGGCAACTAGTCATAAGGGTTGCGCTCGTTGCGGGACTTAACCCAACATCTCACGACACGAGCTGACGACAACCATGCACCACCTGTCATTTTGCCCCCGAAGGGGAAACCTGATCTCTCAGGTGATCAAAAGATGTCAAGACCTGGTAAGGTTCTTCGCGTTGCTTCGAATTAAACCACATGCTCCACCGCTTGTGCGGGCCCCCGTCAATTCCTTTGAGTTTCAACCTTGCGGTCGTACTCCCCAGGCGGAATGCTTAATGCGTTAGCTGCGGCACTGAAGGGCGGAAACCCTCCAACACCTAGCATTCATCGTTTACGGCATGGACTACCAGGGTATCTAATCCTGTTCGCTACCCATGCTTTCGAGCCTCAGCGTCAGTTACAGACCAGACAGCCGCCTTCGCCACTGGTGTTCTTCCATATATCTACGCATTTCACCGCTACACATGGAGTTCCACTGTCCTCTTCTGCACTCAAGTTTCCCAGTTTCCGATGCACTTCCTCGGTTAAGCCGAGGGCTTTCACATCAGACTTAAAAAACCGCCTGCGCTCGCTTTACGCCCAATAAATCCGGATAACGCTTGCCACCTACGTATTACCGCGGCTGCTGGCACGTAGTTAGCCGTGGCTTTCTGGTTGGATACCGTCACGCCGACAACAGTTACTCTGCCGACCATTCTTCTCCAACAACAGAGTTTTACGACCCGAAAGCCTTCTTCACTCACGCGGCGTTGCTCCATCAGACTTGCGTCCATTGTGGAAGATTCCCTACTGCTGCCTCCCGTAGGAGTTTGGGCCGTGTCTCAGTCCCAATGTGGCCGATCAACCTCTCAGTTCGGCTACGTATCATTGCCTTGGTGAGCCGTTACCTCACCAACTAGCTAATACGCCGCGGGTCCATCCAAAAGCGATAGCTTACGCCATCTTTCAGCCAAGAACCATGCGGTTCTTGGATTTATGCGGTATTAGCATCTGTTTCCAAATGTTATCCCCCACTTAAGGGCAGGTTACCCACGTGTTACTCACCCGTCCGCCACTCGTTCAAAATTAAATCAAGATGCAAGCACCTTTCATTAATCAGAACTCGTTCGACTTGCAGTATAGGCA |
| LPF1 | AATAACGGCTCGCTGCCTAGAAGGGTTACTCCACCGGCTTCGGGTGTTACAAACTCTCATGGTGTGACGGGCGGTGTGTACAAGGCCCGGGAACGTATTCACCGCGGCGTGCTGATCCGCGATTACTAGCGATTCCGACTTCGTGTAGGCGAGTTGCAGCCTACAGTCCGAACTGAGAATGGCTTTAAGAGATTAGCTTGACCTCGCGGTCTCGCAACTCGTTGTACCATCCATTGTAGCACGTGTGTAGCCCAGGTCAAAAGGGGCATGATGATTTGACGTCATCCCCACCTTCCTCCGGTTTGTCACCGGCAGTCTTACTAGAGTGCCCAACTAAATGCTGGCAACTAGTCATAAGGGTTGCGCTCGTTGCGGGACTTAACCCAACATCTCACGACACGAGCTGACGACAACCATGCACCACCTGTCATTTTGCCCCCGAAGGGGAAACCTGATCTCTCAGGTGATCAAAAGATGTCAAGACCTGGTAAGGTTCTTCGCGTTGCTTCGAATTAAACCACATGCTCCACCGCTTGTGCGGGCCCCCGTCAATTCCTTTGAGTTTCAACCTTGCGGTCGTACTCCCCAGGCGGAATGCTTAATGCGTTAGCTGCGGCACTGAAGGGCGGAAACCCTCCAACACCTAGCATTCATCGTTTACGGCATGGACTACCAGGGTATCTAATCCTGTTCGCTACCCATGCTTTCGAGCCTCAGCGTCAGTTACAGACCAGACAGCCGCCTTCGCCACTGGTGTTCTTCCATATATCTACGCATTTCACCGCTACACATGGAGTTCCACTGTCCTCTTCTGCACTCAAGTTTCCCAGTTTCCGATGCGCTTCCTCGGTTAAGCCGAGGGCTTTCACATCAGACTTAAAAAACCGCCTGCGCTCGCTTTACGCCCAATAAATCCGGATAACGCTTGCCACCTACGTATTACCGCGGCTGCTGGCACGTAGTTAGCCGTGGCTTTCTGGTTGGATACCGTCACGCCGACAACAGTTACTCTGCCGACCATTCTTCTCCAACAACAGAGTTTTACGACCCGAAAGCCTTCTTCACTCACGCGGCGTTGCTCCATCAGACTTGCGTCCATTGTGGAAGATTCCCTACTGCTGCCTCCCGTAGGAGTTTGGGCCGTGTCTCAGTCCCAATGTGGCCGATCAACCTCTCAGTTCGGCTACGTATCATCGCCTTGGTGAGCCATTACCTCACCAACTAGCTAATACGCCGCGGGTCCATCCAAAAGCGATAGCTTACGCCATCTTTCAGCCAAGAACCATGCGGTTCTTGGATCTATGCGGTATTAGCATCTGTTTCCAAATGTTATCCCCCACTTAAGGGCAGGTTACCCACGTGTTACTCACCCGTCCGCCACTCGTTCCATGTTGAATCTCGGTGCAAGCACCGATCATCAACGAGAACTCGTTCGACTTGCAGTTCC |
| LRA3 | CAGAACGGTTCGGGTGTTACAACTCTCATGGTGTGACGGGCGGTGTGTACAAGGCCCGGGAACGTATTCACCGCGGCGTGCTGATCCGCGATTACTATCTATTCCGACTTCGTGTAGGCGAGTTGCAGCCTACAGTCCGAACTGAGAATGGCTTTAAGAGAATAGCTTGACCTCGCGGTCTCGCAACTCGTTGTACCATCCATTGTAGCACGTGTGTAGCCCAGGTCATAAGGGGCATGATGATTTGACGTCATCCCCACCTTCCTCCGGTTTGTCACCGGCAGTCTTACTAGAGTGCCCAACTAAATGCTGGCAACTAGTCATAAGGGTTGCGCTCGTTGCGGGACTTAACCCAACATCTCACGACACGAGCTGACGACAACCATGCACCACCTGTCATTTTGCCCCCGAAGGAGAAACCTGATCTCTCAGGTGATCAAAAGATGTCAAGACCTGGTAAGGTTCTTCGCGCTGCTTCGAATTAAACCCACATGCTCCACCGCTTGTGCGGGCCCCCGTCAATTCCTTTGAGTTTCAACCTTGCGGTCGTACTCCCCAGGCGGAATGCTTAATGCGTTAGCTGCGGCACTGAAGGGCGGAAACCCTCCAACACCTAGCATTCATCGTTTACGGCATGGACTACCAGGGTATCTAATCCTGGTGCGCTACCCATGCTTTCGAGCCTCAGCGTCAGTTACAGAACCAGACAGCCGCCTTCGCCACTGGTGTTCTTCCATATATCTACGCATTTCACCGCTACACATGGAGTTCCACTGTCCTCTTCTGCACTCAAGTTTCCCAGTTTCCGATGCACTTCCTCGGTTAAGCCGAGGGCTTTCACATCAGACTTAAAAAACCGCCTGCGCTCGCTTTACGCCCAATAAATCCGGATAACGCTTGCCACCTACGTATTACCGCGGCTGCTGGCACGTAGTTAGCCGTGGCTTTCTGGTTGGATACCGTCACGCCGACAACAGTTACTCTGCCGACCATTCTTCTCCAACAACAGAGTTTTACGACCCGAAAGCCTTCTTCACTCACGCGGCGTTGCTACATCAGACTTGCGTCCATTGTGGAAGATTCCCTACTGCTGCCTCCCGTAGGAGTTTGGGCCGTGTCTCAGTCCCAATGTGGCCGATCAACCTCTCAGTTCGGCTACGTATCATTGCCTTGGTGAGCCGTTACCTCACCAACTAGCTAATACACAGCGGGTCCATCCAAAAGCGATAGCTT |

Supplementary data 3 – Sequences of the V1–V9 regions of the 16S rRNA gene from the 11 isolated strains.

| **Isolated strains names** | **Genus** | **Specie** | **Query Length** | **Query Cover** | **Sequence Identity** |
| --- | --- | --- | --- | --- | --- |
| BLA1 | *Bifidobacterium* | *longum* | 1387 | 99% | 99.78% |
| BBB1 | *Bifidobacterium* | *breve* | 1401 | 99% | 99.86% |
| BDB1 | *Bifidobacterium* | *dentium* | 1405 | 100% | 99.71% |
| BBC1 | *Bifidobacterium* | *breve* | 1367 | 99% | 99.85% |
| BBD1 | *Bifidobacterium* | *breve* | 1387 | 99% | 99.93% |
| BLE1 | *Bifidobacterium* | *longum subsp. suillum* | 1398 | 100% | 100% |
| BPE1 | *Bifidobacterium* | *pseudocatenulatum* | 1364 | 100% | 99.78% |
| BBF1 | *Bifidobacterium* | *breve* | 1367 | 99% | 99.70% |
| LRB1 | *Lacticaseibacillus* | *rhamnosus* | 1453 | 100% | 99.72% |
| LPF1 | *Lacticaseibacillus* | *paracasei* | 1447 | 99% | 99.79% |
| LRA3 | *Lacticaseibacillus* | *rhamnosus* | 1233 | 99% | 98.94% |

Supplementary data 4 – Summary of 16S rRNA BLASTn matches for 11 bacterial isolates.

| **Strain** | **BLA1** | **LRB1** | **BBD1** | **BLE1** |
| --- | --- | --- | --- | --- |
| Coverage | 213 | 180 | 83 | 93 |
| Completeness | 99,97 | 99,91 | 99,99 | 99,98 |
| Contamination | 0,06 | 0,17 | 0,08 | 0,42 |
| No chimerism | True | True | True | True |
| Classification | *Bifidobacterium longum subsp. longum* | *Lacticaseibacillus rhamnosus* | *Bifidobacterium breve* | *Bifidobacterium longum subsp. longum* |
| Closest genome reference | GCF_000196555.1 | GCF_900636965.1 | GCF_001025175.1 | GCF_000196555.1 |
| Closest genome ANI | 98,44 | 99,62 | 98,65 | 98,64 |
| Contigs | 1 | 1 | 1 | 3 |
| Largest contig | 2 378 534 | 2 945 769 | 2 334 692 | 2 572 355 |
| Total length | 2 378 534 | 2 945 769 | 2 334 692 | 2 611 296 |
| GC (%) | 60,16 | 46,74 | 58,64 | 60,41 |
| N50 | 2 378 534 | 2 945 769 | 2 334 692 | 2 572 355 |
| N90 | 2 378 534 | 2 945 769 | 2 334 692 | 2 572 355 |
| L50 | 1 | 1 | 1 | 1 |
| L90 | 1 | 1 | 1 | 1 |
| # N's per 100 kbp | 0 | 0 | 0 | 0 |
| Plasmids | 0 | 0 | 0 | 2 |
| Prophages | 0 | 1 questionable, 1 intact | 0 | 2 intact |
| Insertion Elements | 7 | 0 | 2 | 15 |
| Genes | 1 989 | 2 802 | 2 320 | 1 979 |
| CDS | 1 924 | 2 725 | 2 237 | 1 918 |
| rRNA | 8 | 15 | 8 | 6 |
| tRNA | 56 | 61 | 74 | 54 |
| tmRNA | 1 | 1 | 1 | 1 |
| VFDB genes | *groEL2* | *lisR, tufA, hasC* | *groEL2, rfbB* | *groEL2* |
| Toxins | *tlyC, hlyIII* | *tlyC, eta, hlyIII* | *tlyC, hlyIII* | *tlyC, hlyIII* |
| Resistence genes | *ileS, mef, rpoB* | *mef, lsa* | *ileS, mef, rpoB, tet(O)* | *ileS, mef, rpoB* |
| Resistence genes in MGE | - | - | - | - |
| Cas system | none | none | Subtype I-C | Subtype II-C |
| CRISPR arrays | 0 | 0 | 2 | 2 |
| Bile salt deconjugation | *cbh* | *-* | *cbh* | *cbh* |
| Biogenic amine formation | - | *odcI* | - | - |

Supplementary data 5 – Genomic characteristics of BLA1, BLE1, BBD1 and LRB1 after whole genome analysis.
